# Expressive pragmatic language in mood and psychotic disorders: a systematic review and meta-analysis

**DOI:** 10.1038/s41537-026-00733-2

**Published:** 2026-02-14

**Authors:** Fiona Meister, Martin Sellier Silva, Gleb Melshin, Chaimaa El Mouslih, Farida Zaher, Roozbeh Sattari, Hsi T. Wei, Neyra Mekideche, Valentina Bambini, Alban Voppel, Lena Palaniyappan

**Affiliations:** 1 Department of Psychiatry, McGill University, Montreal, QC Canada; 2 Douglas Mental Health University Institute, Montreal, QC Canada; 3 Department of Medicine, McGill University, Montreal, QC Canada; 4 Department of Psychology, McGill University, Montreal, QC Canada; 5 Laboratory of Neurolinguistics and Experimental Pragmatics (NEPLab), University School for Advanced Studies IUSS, Pavia, Italy; 6 Robarts Research Institute, Lawson Health Research Institute, London, ON Canada; 7 Department of Psychiatry, Schulich School of Medicine and Dentistry, Western University, London, ON Canada

**Keywords:** Psychosis, Biomarkers

## Abstract

Pragmatic language impairments—difficulties using language effectively in social contexts—are common in adults suffering from severe mental illnesses (SMIs) such as schizophrenia spectrum disorders (SSD), major depressive disorder (MDD), and bipolar disorder (BD). These impairments hinder social functioning and recovery but have been explored most widely using comprehension tasks, with pragmatic production being poorly described. We undertook a systematic review and meta-analysis of studies assessing expressive pragmatic language in adults with SMIs versus healthy controls. 18 items were tested, including Coherence, Cohesion, Gricean maxims, figurative language, Prosody, and Turn-Taking. The searches were PRISMA-compliant and were conducted in PubMed and Scopus. 51 studies were included; 28 were meta-analyzed. Results showed significant impairments in Cooperativity, Anaphora and Cohesion, moderate impairments in Coherence, and low impairments in Metaphor. No significant moderator was detected. Our results emphasize the need for standardized pragmatic testing and intervention for language production in clinical settings.

## Introduction

Language in social interactions requires more than following grammar rules and the literal meaning of words: It is also highly dependent on context. The ability to use language in context is referred to as pragmatics^1^ and plays a significant role in mental health. Impairments in social communication, resulting from pragmatic difficulties, are prevalent across various mental health conditions^2,3^. For instance, in Schizophrenia Spectrum Disorders (SSD), pragmatic impairments are said to affect 70–80% of individuals^4^. Difficulties organizing one’s speech coherently might also be observed in Major Depressive Disorder (MDD)^2^. In addition, the presence of pragmatic breakdowns in understanding and following rules of conversation in social settings also increases the risk of developing MDD^5,6^. Mood states in Bipolar Disorder (BD) are characterized by violations of the pragmatic maxim of quantity, with individuals saying either too much or too little depending on their manic or depressive phase. Despite the importance of pragmatics for SSD, MDD, and BD—three serious mental illnesses (SMI)^7^ commonly jointly investigated in psychiatric (speech) studies^8,9^,—there remains a significant knowledge gap. First, a large number of studies conducted so far assess pragmatic abilities through comprehension tasks^10^ (e.g., understanding of figurative language) rather than expressive tasks^11,12^ (e.g., adherence to conversational rules). Second, among the studies that do focus on expressive pragmatic tasks, a large body only does so at a superficial level by investigating “discourse coherence” at large, without providing information about its underlying mechanisms, such as anaphora production. In addition, studies focusing on other aspects of expressive pragmatics, such as the use of figurative language or adherence to conversational rules (cooperativity), are rare. As a result, it remains unclear which expressive aspects of pragmatics beyond/below coherence are affected in SMIs and to what extent. This knowledge is essential not only to improve the assessment of social communication deficits but also to devise measurements that can sensitively track the effect of interventions that address pragmatic impairments.

Studying expressive pragmatics in SMIs is crucial for 5 key reasons. (i) Expressive pragmatic skills are fundamental in interpersonal relationships. Deficits are associated with awkwardness^13^, social rejection, and overall poor community integration, which in turn can aggravate SMIs symptoms^14^. (ii) Expressive pragmatic impairments are a major component of the praecox feeling in clinical settings (i.e., bizarreness perceived by a therapist when interacting with SSD patients), which can create a barrier between the patient and clinician^15,16^. This can lead to exclusion from psychotherapy and affect therapeutic alliance, which in turn can worsen long term outcome^17^. (iii) Vocational success, a cornerstone in recovery^18^ depends on the ability to communicate pragmatically (see^19,20^ for further discussion). (iv) Certain risk factors for SMIs, such as immigration^21,22^ may exacerbate expressive pragmatic challenges^23^, highlighting the need for tailored support. (v) Some key symptoms of SMIs may be directly related to expressive pragmatic impairments (e.g., poor coherence resulting in disorganized speech, pauses/prosodic deficits contributing to psychomotor retardation and negative symptoms, reduced speech output), suggesting that addressing these impairments could lead to improved symptom management. In summary, social interactions are paramount for psychosocial recovery^24^, and effective pragmatic skills are essential for navigating these interactions successfully^20,25^. Understanding the nature and degree of expressive pragmatic impairments in individuals with SMIs is an essential first step to improving treatment outcomes linked to these deficits.

There are several approaches to assess pragmatics. First, using checklists or questionnaires together with input from those who interact regularly with the individual being assessed (e.g., Pragmatic Language Skills Inventory^26^ or the Children’s Communication Checklist^27^). While this is very informative for children where parents or teachers can provide sufficient details, such assessments are often incomplete for adults with SMIs. Second, using standardized assessments such as the Assessment Battery for Communication (ABaCo)^28^ or the Test for the Assessment of Pragmatic Abilities and Cognitive Substrates (APACS)^29^. These assessments are increasingly applied in SMIs, although it must be noted that they are not restricted to production as they also include comprehension tasks. Third, parsing the elements representing pragmatic production from spontaneous speech and prompted narratives. In the current review, we focus on the latter two approaches given their applicability to expressive pragmatic impairment in adults with SMIs.

Our aim is to identify and quantify expressive pragmatic impairments. Given the expected variability in the severity and presentation of these impairments across studies, our secondary aim is to explore potential moderators (e.g., diagnosis, sex, age) that may influence the relationship between SMIs and expressive pragmatic impairments. By addressing these issues, we seek to identify critical research gaps that hinder the consideration of pragmatics in clinical practice and to provide grounds for rehabilitative programs addressing expressive pragmatic impairments associated with SMIs.

## Methods

### Selecting variables to study pragmatics

Given the breadth of expressive pragmatics, defining the scope of this review is a key challenge. Even in disorders where expressive pragmatic impairments are the cornerstone (i.e. Social Pragmatic Communication Disorder), no consensus has been reached regarding which pragmatic component should be assessed^30,31^.

In accordance with previous influential work in psychiatry^1^ and several pragmatic assessment tools (e.g. Assessment of Pragmatic Abilities and Cognitive Substrates [APACS]^29^, Montreal Protocol for the Evaluation of Communication [MEC]^32^) we used Sperber & Wilson’s (2005)^33^ widely accepted two-fold definition of pragmatics to guide our study: (i) The interaction of contextual factors with literal meaning in utterances interpretation. In other words, how do interpretations of similar linguistic structures vary based on context (e.g., In I gave you my dessert, how does the meaning of *I*, *my*, and *you* vary based on who the speaker and listener are)? This includes inferring the speaker’s meaning, especially when language is not used literally. (ii) The use of language as a social phenomenon to achieve a shared communicative goal. This involves communicating the right amount of information to be understood and organizing one’s speech coherently.

Thus, in line with previous works^34^, we incorporated the above concepts and considered a phenomenon to be pragmatic in nature if it bridges the gap between the literal and intended by either: (i) Figurative language (i.e. Expression of humorous content, Idioms Usage,Irony production, and Metaphor usage); (ii) Discursive phenomena dependent on either grammar (i.e. Anaphoric reference production, Coherence, Cohesion, and Appropriate thematic organization, Deictic expressions production, and Appropriate use of presuppositions) or social conventions (i.e. Cooperativity principles observance, Generation of implicit meaning, Conveying of explicatures/implicatures, Appropriate use of pauses, Prosodic usage, Observance of social norms, Production of speech acts, and Management of conversational turns). These phenomena are largely employed in everyday conversation^35,36^, making them a core aspect of expressive pragmatic competence (“meaning minus semantics”)^26^. Moreover, recent studies have highlighted the presence of large effect-size deficits in figurative expressions that can be rectified with focused remediation approaches^36^. Finally, non-linguistic features such as eye contact and hand gestures were not considered here.

Guided by these principles, we built a large list of expressive pragmatic features that defined the scope of our search process. In generating this list, we selected features that observed the following conditions: (i) Distinctiveness: Each feature must represent a distinct aspect of expressive pragmatic communication and is not measured by another feature under a different name. When a component is hierarchically nested within another (e.g., Cohesion within Coherence), the lower-level component is included only if it captured finer-grained elements not encompassed by its higher-level parent (e.g. Cohesive devices not reflected by Coherence); (ii) Operationalizable: The features are not merely descriptive, but can be measured as a variable by counting or grading; (iii) Coverage Adequacy: The final set of components should be as exhaustive as possible in its coverage of relevant aspects of expressive pragmatic impairments.

The final list was consistent with two main bodies of resources relevant to our question: (i) Previous studies investigating pragmatic impairments in SMIs or developmental/acquired neurological disorders affecting pragmatic communication, such as Autism Spectrum Disorder, aphasia, right hemisphere damage^37–39^; (ii) Canonical linguistics literature covering pragmatics^40–45^. Therefore, this work aimed to investigate the expressive pragmatic components outlined in Table 1 in the SMIs literature without prior assumption about their existence/frequency. Since the definitions/boundaries of some of these components might be subject to debate, additional information regarding our categorization process is provided in Section S1 of the supplementary material.

**Table 1:** List and definitions of expressive pragmatic features investigated.

Expressive Pragmatic Component	Definition
**Anaphoric Reference Production (Anaphora)**	Ability to use linguistic expressions that refer back to previously mentioned elements in discourse/context^43^.
**Coherence**	Ability to use logical and meaningful connections within and across sentences to create a unified text in context^128,129^.
**Cohesion**	Ability to use linguistic elements (e.g., conjunctions, pronouns, repeated words) to link parts of discourse together^43^.
**Deictic Expressions Production (Deixis)**	Ability to use words or phrases (e.g., ‘this’, ‘that’, ‘here’, ‘there’) that depend on context for interpretation^130^.
**Cooperativity Principles Observance**	Ability to observe and use the principles guiding effective conversation and language use, including relevance, clarity, and informativeness based on Grice’s Maxims^42^.
**Expression of Humorous Content (Humor)**	Ability to use language to create amusement, often involving incongruity, surprise, or wordplay^131^.
**Idioms Usage**	Ability to use fixed expressions whose meanings cannot be inferred from the individual words^132^.
**Generation of Implicit Meaning (Inferences)**	Ability to generate utterances that require the listener to derive implicit meanings via Inferences, rather stating everything explicitly^42^.
**Conveying of Explicatures/Implicatures**	Ability to generate utterances that convey explicatures (context-based enrichment of logical forms) and Implicatures (implicated meanings)^133^.
**Irony Production**	Ability to use rhetorical devices that convey a meaning that is opposite to the literal interpretation^42^.
**Metaphor Usage**	Ability to use figurative expressions that involve a conceptual shift, typically defined as the description of one element in terms of another^134^.
**Appropriate Use of Pauses**	Ability to use breaks or silences in speech that contribute to meaning, structure, or emphasis^135^.
**Use of Presuppositions**	Ability to produce utterances that encode background assumptions about the listener’s knowledge or belief^136^.
**Prosodic Usage (Prosody)**	Ability to use rhythmic and intonational aspects of speech that influence meaning beyond words. Of the various prosodic features, we only focused on non-affective Prosody i.e., pragmatic Prosody as defined by^137^, and discussed in depth by^120^.
**Observance of Social Norms**	Ability to respect culturally and socially accepted conventions that govern language use (e.g., politeness)^138^.
**Production of Speech Acts**	Ability to use communicative acts (e.g., requests, apologies, promises) that perform functions in interaction that may go beyond the literal meaning (e.g., “Can you hold the door for me?”)^45^.
**Appropriate Thematic Organization**	Ability to structure discourse around coherent and relevant topics or themes, ensuring that ideas are introduced, maintained, and transitioned in a way that supports overall communicative intent.^43^.
**Management of Conversational Turns (Turn-Taking)**	Ability to manage one’s participation in conversational exchange by appropriately initiating, holding, yielding, or responding to turns^139^.

### Search strategy and selection criteria

This study’s original protocol was registered on the Open Science Framework registry on May 17th, 2024. The study follows the Preferred Reporting Items for Systematic Reviews and Meta-Analyses (PRISMA) guidelines^46^, as well as recent recommendations to mitigate authors’ biases in meta-analysis^47^.

We carried a literature search across two electronic databases: PubMed and Scopus for articles published in English available until June 28th, 2024, with no lower limit on publication date or restriction on the participants’ language. Our search strings were designed to capture the following elements: (1) the mental health conditions of interest and their variants, namely MDD, BD, and SSD; (2) the expressive pragmatic linguistic components as those outlined in Section “Selecting variables to study pragmatics”. See Supplementary Table S1 for complete search strings. To ensure the reliability of our literature search, we used the bibliography of two seed articles^37,48^, which both extensively discuss pragmatic impairments in SMIs, as a benchmark to compare the articles resulting from our search. Minimal initial discrepancies were observed. We thus modified our search string accordingly, which resulted in full coverage of the bibliography from the seed articles. Using the Covidence platform, duplicates were removed before further processing. Following a title and abstract screening, each article was independently screened against the inclusion/exclusion criteria twice independently by the following reviewers: FM, AV, GM, MSS, FZ, and CEM. Conflicts were resolved by consensus before a full-text review was performed.

We included studies that used computational or manual methods to analyze narrative speech, written samples, or employed a multiple-choice test. We excluded studies that focussed only on related constructs such as ToM, Social Cognition, Emotion/Affect production, and/or voice quality without an explicit consideration of expressive pragmatic impairments.

The selected articles had to meet the following inclusion criteria: (i) Enrolling participants aged 18 or older with confirmed SSD or a diagnosis of MDD or BD (see supplementary Table S2 for a comprehensive list of all the variants included); (ii) Including a group of control participants without known psychiatric diagnoses; (iii) Assessing the production of at least one of the pragmatic components listed in Table 1.

Additionally, the following criteria were grounds for exclusion: (i) including mostly participants aged under 18^49^ or above 65^50^; (ii) participants having severe physical^51,52^ and/or psychiatric^53,54^ and/or developmental/pervasive^55–57^ and/or degenerative^58,59^ comorbidities; (iii) focusing on clinical high-risk groups without a confirmed SMIs diagnosis^60^.

### Data extraction

When available, we extracted the following clinical and demographic data (Table 2): Author(s), publication year, diagnosis (grouped under SSD, BD, and MDD), pragmatic component assessed, language of task of administration, sample size, mean age (standard deviation [SD]), symptom severity based on standardized scales [e.g., PANSS, SANS/SAPS, Hamilton Depression Rating Scale], male to female ratio, speech task [e.g., ABaCo, spontaneous speech, etc.]. Mean (SD) were pooled when different variants of a disorder were evaluated in the same study. When multiple measures were reported for the same pragmatic component, we extracted the most representative one. When multiple were equally representative, we reported their pooled mean (SD). Finally, when a single paper (k) reported quantifiable outcomes about different pragmatic components (e.g., Anaphora and Coherence), or clinical population (e.g., SSD and MDD), each outcome was treated as a separate effect size (M)^61^, regardless of the main focus of the paper.

**Table 2:** Description of the included studies.

Study Name	Diagnosis	Expressive Pragmatic Component	PG (n)	HC (n)	Gender		Age (years), mean (SD)		Symptom Severity	Language	Speech Task
					PG (male/female)	HC (male/female)	PG	HC			
Allen & Allen 1985^72^	SSD	Anaphora	19	9	N/A	N/A	50.95 (6.06)	53.00 (2.6)	Standardized Assessment Scale of Krawiecka et al. (1977) Positive: 6.90 (1.73) Negative: 3.11 (1.15)	English	Picture Description
Allen & Allen 1985^72^	SSD	Coherence	19	9	N/A	N/A	50.95 (6.06)	53.00 (2.6)	Standardized Assessment Scale of Krawiecka et al. (1977) Positive: 6.90 (1.73) Negative: 3.11 (1.15)	English	Picture Description
Allen 1984^98^	SSD	Inferences	18	9	N/A	N/A	50.95 (6.06)	53.0 (2.6)	Standardized Assessment Scale of Krawiecka et al. (1977) Positive: 6.90 (1.73) Negative: 3.11 (1.15)	English	Picture Description
Allen 1984^98^	SSD	Thematic Organisation	18	9	N/A	N/A	50.95 (6.06)	53.0 (2.6)	Standardized Assessment Scale of Krawiecka et al. (1977) Positive: 6.90 (1.73) Negative: 3.11 (1.15)	English	Picture Description
Alonso-Sanchez et al. 2022^69^	SSD	Coherence	46	36	36/19	24/11	22.0 (3.6)	21.4 (3.2)	PANSS-8 Positive: 12.1 (3.0) PANSS-8 Negative: 7.4 (4.3) PANSS-8 Total: 25.6 (6.8)	English	Picture Description
Bambini et al. 2016^4^	SSD	Cooperativity	47	35	29/18	14/21	39.74 (10.54)	41.69 (11.61)	PANSS Total: 77.42 (11.51)	Italian	Test Battery (APACS)
Ben Moshe et al. 2024^84^	SSD	Coherence	23	25	23/0	25/0	25.46 (6.39)	33.15 (9.98)	PANSS Positive: 9.21 (3.70) PANSS Negative: 8.26 (3.36) PANSS Total: 17.47 (5.52)	Hebrew	Picture Description
Berardi et al. 2023^110^	SSD	Prosody	20	20	14/6	13/7	41.6 (11.6)	39.3 (12.7)	SANS: 17.3 (11.39) SAPS: 19.32 (15.43)	German	Picture Description
Berardi et al. 2023^110^	MDD	Prosody	20	20	10/10	13/7	41.5 (13.2)	39.3 (12.7)	HAM-D: 6.15 (7.26)	German	Picture Description
Binz & Brüne 2010 ^102^	SSD	Cooperativity	49	29	24/25	10/19	37.76(11.0)	37(8.75)	PANSS positive subscale 17.42 ± 5.02 PANSS negative subscale 20.17 ± 7.39 PANSS total subscale 35.27 ± 7.81	German	Picture Description
Bosco et al. 2019^106^	SSD	Irony	32	32	25/7	25/7	40.17 (10.77)	40.28 (11.16)	PANSS positive: 18.83 (8.89) PANSS negative: 20.28 (9.65) PANSS total: 45.64 (19.02)	Italian	Test Battery (ABaCo)
Cohen et al. 2014^8^	SSD + MDD + BD*Undifferentiated	Prosody	52	30	32/20	13/17	40.38 (12.65)	42.35 (11.99)	BPRS: 1.4 (1.73) SANS: 5.01 (1.15)	English	Spontaneous Speech
Çokal et al. 2018^104^	SSD	Anaphora	30	15	23/7	7/8	44.0 (11.9)	45 (13)	PANSS Positive: 25.5 PANSS Negative: 23.5 PANSS Total: 81.0	English	Picture Description
Çokal et al. 2019^99^	SSD	Pauses	30	15	23/7	7/8	43.21 (12.48)	45 (13)	PANSS (no score available)	English	Retelling Task
Çokal et al. 2023^117^	SSD	Anaphora	31	27	18/13	15/12	36.48 (5)	39 (9)	PANSS Positive: 25.5 PANSS Negative: 23.5 PANSS Total: 81.0	Turkish	Picture Description
Colle et al. 2013^96^	SSD	Irony	17	28	15/2	24/4	36.3 (9.9)	36.2 (9.9)	PANSS Negative: 20.1 (10) PANSS Positive: 21 (9.1) PANSS General: 47.9 (19.9) PANSS Total: 93.7 (40.6)	Italian	Test Battery (ABaCo)
Colle et al. 2013^96^	SSD	Social Norms	17	28	15/2	24/4	36.3 (9.9)	36.2 (9.9)	PANSS PANSS Negative: 20.1 (10) PANSS Positive: 21 (9.1) PANSS General: 47.9 (19.9) PANSS Total: 93.7 (40.6)	Italian	Test Battery (ABaCo)
Colle et al. 2013^96^	SSD	Speech Acts	17	28	15/2	24/4	36.3 (9.9)	36.2 (9.9)	PANSS PANSS Negative: 20.1 (10) PANSS Positive: 21 (9.1) PANSS General: 47.9 (19.9) PANSS Total: 93.7 (40.6)	Italian	Test Battery (ABaCo)
Colle et al. 2013^96^	SSD	Thematic Organisation	17	28	15/2	24/4	36.3 (9.9)	36.2 (9.9)	PANSS PANSS Negative: 20.1 (10) PANSS Positive: 21 (9.1) PANSS General: 47.9 (19.9) PANSS Total: 93.7 (40.6)	Italian	Test Battery (ABaCo)
Colle et al. 2013^96^	SSD	Turn- Taking	17	28	15/2	24/4	36.3 (9.9)	36.2 (9.9)	PANSS PANSS Negative: 20.1 (10) PANSS Positive: 21 (9.1) PANSS General: 47.9 (19.9) PANSS Total: 93.7 (40.6)	Italian	Test Battery (ABaCo)
Corcoran et al. 1996^88^	SSD	Cooperativity	38	13	N/A	N/A	32.61(9.56)	31.46(11.19)	N/A	English	Spontaneous Speech
Corcoran et al. 1996^88^	SSD	Social Norms	38	13	N/A	N/A	32.61(9.56)	31.46(11.19)	N/A	English	Spontaneous Speech
Despot et al. 2021^91^	SSD	Metaphor	5	5	N/A	N/A	N/A	N/A	N/A	Croatian	Retelling Task
Docherty et al. 2003^105^	SSD	Anaphora	48	28	26/22	15/13	36(9)	39(7)	SAPS: 3.63 (2.95) SANS: 1.17 (1.74)	English	Spontaneous Speech
Elvevåg et al. 2007^112^	SSD	Coherence	26	25	19/7	10/15	33.77 (7.63)	35.44 (12.94)	N/A	Norwegian	Spontaneous Speech
Elvevåg et al. 2011^92^	SSD	Metaphor	21	21	N/A	N/A	33.52 (8.10)	33.81 (8.54)	PECC total score: 42.1 (16.3) Positive symptoms: 7.5 (3.9) Negative symptoms: 10.8 (5.4) Depressive symptoms: 9.4 (4.2) Excitement: 7.6 (3.8) Cognitive symptoms: 6.9 (3.1)	Norwegian	Spontaneous Speech
Figueroa-Barra et al. 2022^77^	SSD	Coherence	49	84	50/34	24/25	27.21(6.96)	38.6(15.0)	PANSS Positive: 29.56 (4.80) PANSS Negative: 33.85 (6.01) PANSS General: 68.68 (8.48) PANSS Total: 132.05 (19.37)	Spanish	Spontaneous Speech
Gargano et al. 2022^78^	SSD	Coherence	133	133	80/53	64/69	28.93 (9.05)	33.07 (9.56)	PANSS: 34.95 (8.93)^1^	Italian	Picture Description
Haas et al. 2015^100^	SSD	Implicatures	15	7	N/A	N/A	33.6 (8.2)	28.0 (4.9)	N/A	English	Spontaneous Speech
Harvey 1983^73^	SSD	Cohesion	20	10	6/14	3/7	32.47 (8.21)	30.9 (1.5)	SADS: 29.21 (10.17)	English	Spontaneous Speech
Harvey 1983^73^	BD	Cohesion	20	10	7/13	3/7	30.20 (8.68)	30.9 (1.5)	SADS: 35.96 (6.98)	English	Spontaneous Speech
Harvey 1983^73^	SSD	Anaphora	20	10	6/14	3/7	32.47 (8.21)	30.9 (1.5)	SADS: 29.21 (10.17)	English	Spontaneous Speech
Harvey 1983^73^	BD	Anaphora	20	10	7/13	3/7	30.20 (8.68)	30.9 (1.5)	SADS: 35.96 (6.98)	English	Spontaneous Speech
Harvey 1983^73^	SSD	Deixis	20	10	6/14	3/7	32.47 (8.21)	30.9 (1.5)	SADS: 29.21 (10.17)	English	Spontaneous Speech
Harvey 1983^73^	BD	Deixis	20	10	7/13	3/7	30.20 (8.68)	30.9 (1.5)	SADS: 35.96 (6.98)	English	Spontaneous Speech
Hoffman et al. 1986^79^	SSD	Coherence	39	40	N/A	N/A	26.6	25.7	N/A	English	Spontaneous Speech
Hoffman et al. 1986^79^	BD	Coherence	24	40	N/A	N/A	34.7	25.7	N/A	English	Spontaneous Speech
Howes & Lavelle 2023^101^	SSD	Turn-Taking	20	100	6/14	55/45	41.50 (8.64)	31.10 (9.60)	PANSS positive: 15.80 (6.76) PANSS negative: 9.95 (3.36) PANSS general: 28.41 (10.42)	English	Spontaneous Speech
Jørgensen et al. 2024^113^	SSD	Prosody	17	18	4/13	11/7	21.5	24.4	N/A	Danish	Retelling Task
Just et al. 2020^74^	SSD	Coherence	40	20	22/18	11/9	43.8 (11.42)	43.9 (13.29)	SAPS: 1.00 (0.92) SANS: 1.39 (0.69)	German	Spontaneous Speech
Just et al. 2020^74^	SSD	Anaphora	40	20	22/18	11/9	43.8 (11.42)	43.9 (13.29)	SAPS: 1.00 (0.92) SANS: 1.39 (0.69)	German	Spontaneous Speech
Kauschke et al. 2018^93^	MDD	Metaphor	26	32	10/16	12/20	37.27 (10.59)	37.47 (11.91)	N/A	German	Picture Description
Linscott 2005^89^	SSD	Cooperativity	20	26	16/4	14/12	30.5 (6.3)	27.6 (6.1)	N/A	English	Picture Description
Lucarini et al. 2024^107^	SSD	Turn-Taking	29	29	18/11	13/16	35.52 (12.73)	28.24 (2.95)	PANSS Positive: 15.03 (6.79) PANSS Negative: 22.48 (10.32) PANSS General: 40 (16.04) PANSS Total:77.51 (28.17)	Italian	Spontaneous Speech
Lundin et al. 2023^80^	SSD	Cohesion	32	15	17/15	7/8	41.66 (7.75)	38.87 (8.36)	PANSS Positive: 15.41 (5.88) PANSS Negative: 14.72 (3.75)	English	Spontaneous Speech
Marini et al. 2008^81^	SSD	Coherence	29	48	21/8	35/13	43.4 (13.3)	43.4 (16.8)	PANSS Positive: 28.9 (5.1) PANSS negative: 22.9 (6.6) PANSS General: 52.3 (10.9)	Italian	Picture Description
Marini et al. 2008^81^	SSD	Cohesion	29	48	21/8	35/13	43.4 (13.3)	43.4 (16.8)	PANSS Positive: 28.9 (5.1) PANSS negative: 22.9 (6.6) PANSS General: 52.3 (10.9)	Italian	Picture Description
Mazza et al. 2008^90^	SSD	Cooperativity	38	44	30/8	18/26	38.5 (5.6)	37.4 (3.9)	SANS: 30.78 (5.4) SAPS: 11.38 (4.3)	Italian	Multiple Choice Answer
Morgan et al. 2021^75^	SSD	Anaphora	16	13	13/3	8/5	24.5 (3.7)	26.5 (5.2)	TLI total: 3.5 (2.9) TLI positive: 2.9 (3.0) TLI negative: 0.58 (0.86)	English	Picture Description
Morgan et al. 2021^75^	SSD	Coherence	16	13	13/3	8/5	24.5 (3.7)	26.5 (5.2)	TLI total: 3.5 ± 2.9 TLI positive: 2.9 (3.0) TLI negative: 0.58 (0.86)	English	Picture Description
Morgan et al. 2021^75^	SSD	Thematic Organisation	16	13	13/3	8/5	24.5 (3.7)	26.5 (5.2)	TLI total: 3.5 ± 2.9 TLI positive: 2.9 (3.0) TLI negative: 0.58 (0.86)	English	Picture Description
Palominos et al. 2023^86^	SSD	Anaphora	20	20	12/8	12/8	35.7 (7.5)	32.6 (11.8)	PANSS Positive: 32.4 (4.5) PANSS Negative: 35.4 (5.3) PANSS General: 69.1 (8.0) PANSS Total: 136.8 (15.6)	Spanish	Spontaneous speech
Parola et al. 2018^108^	SSD	Global	26	26	21/5	21/5	40.01 (10.26)	39.85 (10.68)	PANSS Positive: 20.87 (8.79) PANSS Negative: 20.70 (10.38) PANSS Total: 48.64 (19.38)	Italian	Test Battery (ABaCo)
Parola et al. 2020^97^	SSD	Global	32	35	25/7	29/6	40.17 (10.19)	39.46 (10.95)	PANSS Negative: 20.28 (9.65) PANSS Positive: 18.83 (8.89) PANSS Total: 45.64 (19.02)	Italian	Test Battery (ABaCo)
Parola et al. 2023^85^	SSD	Coherence	111	129	65/46	52/59	26.9 (9.57)	26.4 (8.78)	SANS Total: 9.70 (4.39)	Danish	Retelling Task
Parola et al. 2023^85^	SSD	Coherence	25	29	14/11	17/12	29.2 (8.48)	30.7 (7.56)	PANSS Positive: 10.64 (2.72) PANSS Negative: 14.12 (3.90) PANSS Total: 52.87 (10.09)	German	Retelling Task
Parola et al. 2023^85^	SSD	Coherence	26	26	21/5	21/5	27.2 (7.22)	28.5 (7.72)	PANSS Positive: 18.52 (4.36) PANSS Negative: 20.01 (5.25) PANSS Total: 75.72 (10.46)	Chinese	Retelling Task
Pawełczyk et al. 2021^114^	SSD	Social Norms	34	32	18/16	15/17	20.85 (4.27)	20.21 (4.45)	PANSS Positive: 19.44 (7.12) PANSS Negative: 22.29 (4.8) PANSS General: 43.00 (8.49) PANSS Total: 84.61 (15.84)	Polish	Test Battery (RHLB-PL)
Pawełczyk et al. 2019^115^	SSD	Social Norms	20	20	11/9	11/9	19.45 (3.73)	18.25 (3.27)	PANSS Positive: 14 (6) PANSS Negative: 21 (6) PANSS General: 48.02 (9) PANSS Total: 76.50 (16)	Polish	Test Battery (RHLB-PL)
Perlini et al. 2012^82^	SSD	Coherence	30	30	6/24	13/17	39.70 (10.88)	38.53 (12.71)	BPRS total: 42 (14.41)	Italian	Picture Description
Perlini et al. 2012^82^	BD	Coherence	30	30	19/11	13/17	44.83 (9.46)	38.53 (12.71)	BRRS total: 2.73 (4.95)	Italian	Picture Description
Perlini et al. 2012^82^	SSD	Cohesion	30	30	6/24	13/17	39.70 (10.88)	38.53 (12.71)	BPRS total: 42 (14.41)	Italian	Picture Description
Perlini et al. 2012^82^	BD	Cohesion	30	30	6/24	13/17	39.70 (10.88)	38.53 (12.71)	BPRS total: 42 (14.41)	Italian	Picture Description
Rajabzadeh et al. 2024^116^	SSD	Global	43	43	23/20	25/18	40 (1.21)	38 (9.9)	N/A	Farsi	Test Battery (APP)
Rutter 1985^76^	SSD	Anaphora	35	10	6/6	6/6	N/A	N/A	N/A	English	Spontaneous speech
Rutter 1985^76^	MDD + BD	Anaphora	7	10	N/A	6/6	N/A	N/A	N/A	English	Spontaneous Speech
Saccone et al. 2023^109^	SSD	Prosody	4	23	4/0	17/6	40	51.87	N/A	Italian	Spontaneous Speech
Seifpanahi et al. 2023^119^	MDD	Prosody	30	30	0/30	0/30	42.8 (12.48)	43.2 (11.79)	HRS-D 28.47 (6.18)	Farsi	Spontaneous Speech
Sevilla et al. 2018^118^		Anaphora	40	14	24/16	8/6	41.28 (10.88)	39.6 (10.83)	PANSS Positive: 15.48 (6.20) PANSS Negative: 18.23 (5.83) PANSS Total: 75.98 (17.56)	Spanish	Retelling Task
Shafiyan et al. 2022^94^	SSD	Metaphor	15	15	0/15	0/15	47.8 (N/A)	47.4 (N/A)	N/A	Farsi	Retelling Task
Smirnova et al. 2018^95^	MDD	Metaphor	124	77	30/94	16/61	42 (12)	40 (12)	N/A	Russian	Written Sample
Spencer et al. 2021^102^	SSD	Coherence	16	13	13/3	8/5	24.5 (3.7)	26.5 (5.2)	TLI Total: 3.48 (2.9) TLI Positive: 2.88 (3.0) TLI Negative: 0.58 (0.86)	English	Picture Description
Spitzer et al. 1994^111^	SSD	Pauses	32	36	17/15	17/19	35.3 (14.3)	32.1 (13.9)	BPRS-TD: 3.7 (1.06) BPRS Sum Score: 50.6 (12.17)	German	Picture Description
Tagamets et al. 2014^83^	SSD	Coherence	11	11	9/2	6/5	40 (8.95)	47 (9.81)	BPRS Positive: 11.3 (4.8) BPRS Negative: 5.8 (3.6) Total: 28.9 (7.8)	English	Spontaneous Speech
Tan et al. 2021^103^	SSD	Turn-Taking	43	46	26/17	19/27	41.67 (9.89)	38.89 (14.30)	PANSS positive: 13.56 (4.68) PANSS negative: 14.16 (5.44) PANSS Total: 58.21 (15.38)	English	Spontaneous Speech

*PG* Patient Group, *HC* Healthy Controls, *SSD* Schizophrenia Spectrum Disorder, *MDD* Major Depressive Disorder, *BD* Bipolar Disorder, *SD* standard deviation, *N/A* not applicable, *PANSS* Positive and Negative Syndrome Scale, *SANS* Scale for the Assessment of Negative Symptoms, *SAPS* Scale for the Assessment of Positive Symptoms, *HRS-D* Hamilton Depression Rating Scale, *HAM-D* Hamilton Depression Rating Scale, *BPRS* Brief Psychiatric Rating Scale, *PECC* Psychosis Evaluation tool for Common use by Caregivers, *SADS* Social Avoidance and Distress Scale, *TLI* Thought and Language Index, *APACS* Assessment of Pragmatic Abilities and Cognitive Substrates, *TAT* Thematic Apperception Test, *ABaCo* Assessment Battery for Communication, *RHLB-PL* Right Hemisphere Language Battery Polish Version, *APP* Adult Pragmatics Profile.

### Quality assessment

The quality of each study was assessed using the Newcastle-Ottawa scale^62^, which is widely used in psychiatric meta-analysis^63^. The scale was adapted in line with our two other prior linguistic meta-analyses^64,65^. Thus, the following indices were evaluated: Representativeness of the sample, selection of controls, patients’ disorder assessment validity, confounding factors controlled and matched, assessment of outcome, blinding, and statistical data. Each item was independently rated on a scale of 0–2 for a total of 14 points and disagreements were resolved by discussion.

### Data analysis

Expressive pragmatic components were meta-analyzed if at least m = 5 effect-size estimates^66^ (Hedges’ g) could be obtained. Statistical analyses were performed using the JASP 0.19.2.0 package (R-based)^67^.

Consistent with prior work from our group^65^, effect sizes were pooled using Bayesian model-averaged (BMA) meta-analysis via the metaBMA R package, as implemented in JASP^68^ with default priors for heterogeneity (Inverse-Gamma [1, 0.15]) and effect size (Cauchy [0, 0.707]). This approach estimates a model-averaged effect size by weighing evidence across competing models that incorporate both the magnitude and heterogeneity of effects. Evidence for group differences was interpreted according to Bayes Factor thresholds: weak (BF₁₀ = 1–3), moderate (3–10), strong (10–30), very strong (30–100), and extreme (BF₁₀ > 100)^65^.

If BMA revealed a significant level of heterogeneity between studies within the same pragmatic component, meta-regression analyses were performed to identify potential moderators^61^. Potential moderators included: age and sex of participants, language of assessment (categorized as “English” versus “non-English”), SMIs diagnosis, quality of study, symptom severity, and method of analysis (manual vs. computational). In line with our prior work^65^, only the most frequent scales, namely PANSS (and its derivatives), SAPS, and SANS were included after conversion of severity scores using the min-max normalization. Given the lack of data in the meta-analyzed studies on symptom severity in MDD and BD, symptom severity was only analyzed as a potential moderator for SSD^65^. Finally, Publication bias was evaluated using Robust Bayesian Model-Averaged meta-analysis (RoBMA), implemented in JASP^67^, with default priors on the effect size (Normal[0, 1]), heterogeneity (Inverse-Gamma[1, 0.15]), and publication bias components (selection models and PET/PEESE using Cauchy[0, 1] priors).

Finally, we used Bayesian average model to pool the 5 domain-specific Hedges’ g estimates to assess the overall effect of SMIs on pragmatic production.

### Ethics approval and consent to participate

This study involved secondary analyses of previously published data and did not involve the collection of new data from human participants. As such, ethical approval and informed consent were not required.

## Results

### Study selection

**Fig. 1:**
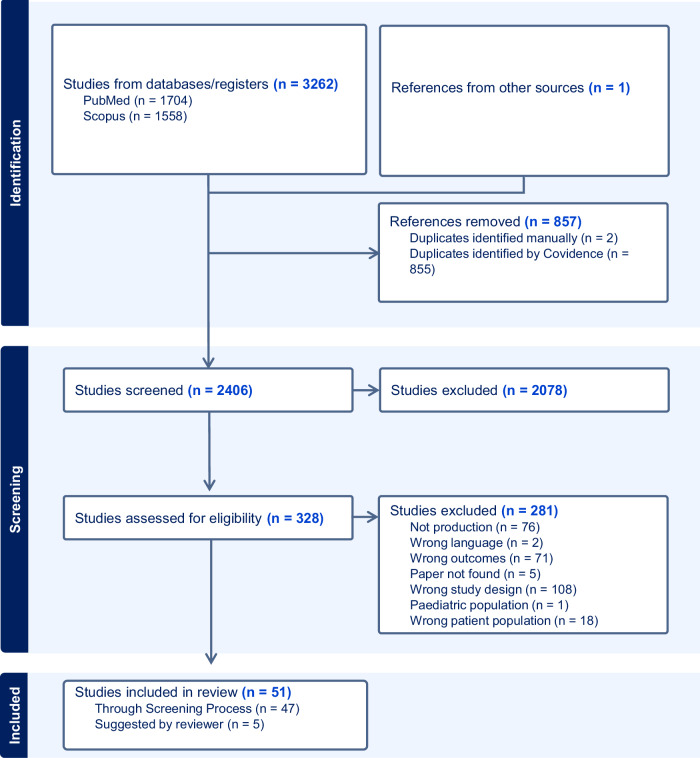
PRISMA 2020 flow diagram for the systematic review and meta-analysis of psychotic and mood disorders.

Our search strings yielded 3263 results (1 article added manually^69^). Of these, 857 were duplicates (2 were identified manually^70,71^), leaving a total of 2406 unique studies. 329 articles remained after title and abstract screening and 281 were excluded upon full-text analysis. In addition, 4 studies were suggested to us during the revision process. In total, 51 papers met full inclusion criteria and 28 could be included in the meta-analysis^4,69,72–97^ (see Fig. 1).

### Study characteristics

A summary of the included papers is displayed in Table 2. Papers reporting more than one outcome of interest (pragmatic component/population) are outlined as separate studies. The papers’ publication date ranged between 1983 and 2024, accounting for a total of 1892 unique patients and 1686 unique healthy controls. The weighted mean age (SD) across papers were 35.51(11.85) for patients and 35.00(12.25) for healthy controls, with no difference in distribution among patients and control group (paired t = –0.10, *p* = 0.92). The most frequent language of task administration was English (k = 19)^8,69,72,73,75,76,79,80,83,88,89,98–105^, Italian (k = 11)^4,78,81,82,90,96,97,106–109^, and German (k = 6)^74,85,87,93,110,111^. K = 48 studies reported data on SSD^8,29,69,72–92,94,96–118^, k = 4 on MDD^93,95,110,119^, k = 3 on BD^73,79,82^, k = 1 considered MDD and BD as one group^76^, and k = 1 considered all three SMIs as one group^8^. Out of those, k = 5 studies investigated more than one SMIs^73,76,79,82,110^. Of the 18 pre-selected pragmatic features listed in Section “Selecting variables to study pragmatics”, studies on SMIs covered 13 features, namely Coherence (k = 12, M = 15)^69,72,74,75,77–79,82–85,88,102,112^, Anaphora (k = 10, M = 11)^72–76,86,104,105,117,118^, Cooperativity (k = 5, M = 5)^50,87–90^, Metaphor (k = 5, M = 5)^91–95^, Cohesion (k = 2, M = 6)^73,80–82^, Prosody (k = 5, M = 3)^8,109,110,113,119^, Social Norms (k = 4, M = 4)^88,96,114,115^, Turn-Taking (k = 4, M = 4)^96,101,103,120^, Irony production (k = 2, M = 2)^99,111^, Pauses (k = 2, M = 2)^99,111^, thematic organization (k = 2, M = 2)^96,98^, Implicatures production (k = 1, M = 2)^100^, Inferences production (k = 1, M = 1)^98^, and Speech Acts (k = 1, M = 1)^96^. Finally, (k = 3, M = 3)^97,108,116^ reported Global measures of pragmatic production^***^. ^108^ reported a global score for (pragmatic) linguistic production from the *Assessment battery for communication (ABaCo)*. This score was not broken down into narrower components. In^97^ the distinction between production and comprehension or between multiple productive components was not clear.^116^ reported a global pragmatic verbal score from the *Adult Pragmatics Profile (APP)*. This score was not broken down into narrower components. Out of these, 5 features met the prespecified criteria of (M ≥ 5) effect sizes to be meta-analyzed, namely Coherence, Cooperativity, Cohesion, Metaphor, and Anaphora. Although, k = 5 independent studies for Discourse Prosody were identified, only M = 3 effect-size estimates could be obtained, as two studies did not have enough information to extract effect sizes M^110,113^. In addition, k = 2 studies^102,112^ did not provide sufficient information to calculate the effect size M and were thus excluded from the Coherence meta-analysis. Regarding quality assessment of the meta-analyzed studies, overall methodological quality was very good, with a mean score of 10.75 (SD = 2.43) and a range of 5–14 (on a 14-point scale). Using standard interpretive thresholds for cross-sectional studies (“very good”: 10–14; “satisfactory”: 6–10; “unsatisfactory”: 0–6), quality scores by pragmatic domain showed similar patterns. Coherence had the highest quality ratings, with a mean score of 11.4 (SD = 2.11) and a range of 7–14, followed by Cohesion with a mean score of 11.7 (SD = 2.16) and a range of 8–13, and Anaphora with a mean score of 10.1 (SD = 3.18) and a range of 5–13. Cooperativity had a mean score of 9.8 (SD = 2.05) and a range of 8–12, and Metaphor had a mean score of 9.8 (SD = 2.68) and a range of 6–12, falling at the boundary between “satisfactory” and “very good.” Overall, these results indicate that the included studies were of consistently high methodological quality across all pragmatic conditions. The quality score of each study included in the meta-analyses is displayed in Table S3 of the supplementary material. Regarding methods of analysis, most studies investigating Anaphora employed manual methods using human raters (k = 8), whereas k = 2^75,86^ used automated approaches. For Cohesion, k = 2^80,82^ studies employed computational methods and k = 2^73,81^ manual methods. Regarding Coherence measures, k = 7 ^83,88,89,91,97–99^ studies employed automated methods, whereas k = 6^72,73,78,79,81,82^. Finally, Cooperativity and Metaphor were always measured based on the judgements made by human raters.

### Data availability

Out of the 47 studies, the language of task administration was reported or could be inferred in all studies. Mean age and SD were missing in k = 2 studies^76,91^. Sex distribution was missing in k = 8 studies^72,76,79,88,91,92,98,100^. Symptom severity was missing in k = 13 studies^76,79,88,89,91,93–95,100,109,112,113,116^. In general, PANSS, SAPS, and SANS were the most commonly used scales for symptoms. Therefore, in the meta-analysis, symptom severity score was only included in the meta-regression analysis for these 3 scales using min-max normalization^65^.

### Meta-analytical results

**Fig. 2:**
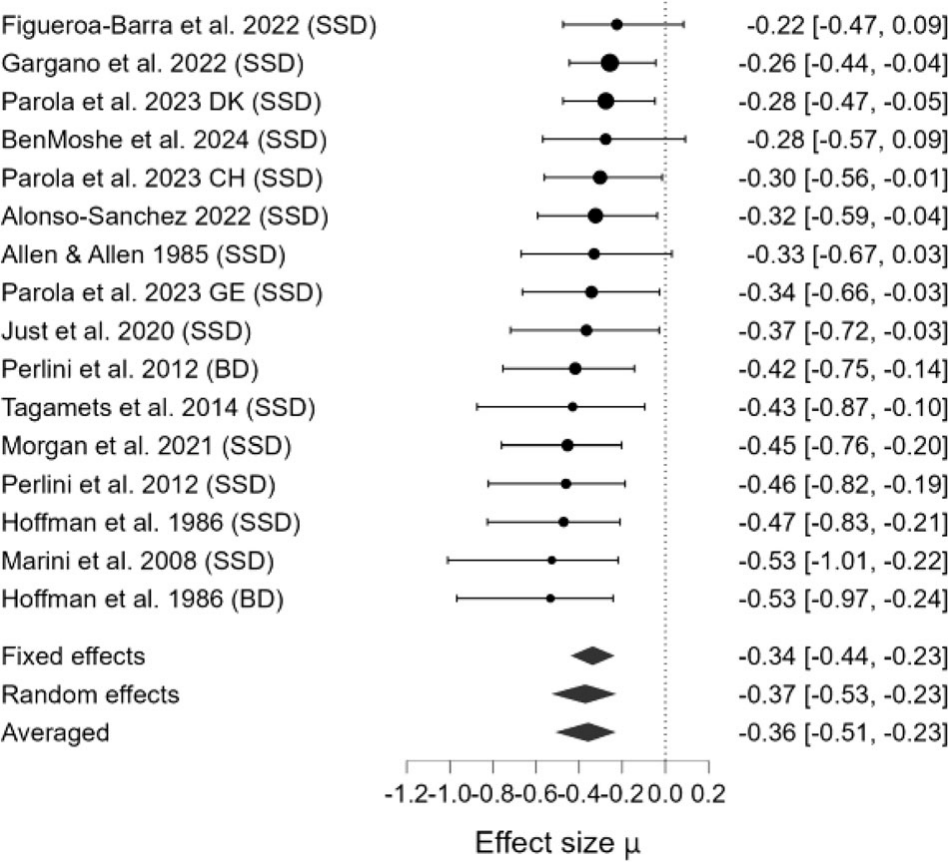
Forest plot for meta-analysis of Coherence impairments in mental health disorders. Bayesian Model Averaged estimates of group differences in Coherence impairment. Estimated (not observed) Hedges’ g values (patients >controls) are given along with 95% credible intervals in parentheses. Negative values indicate that patients performed worse on Coherence metrics than controls, while positive values indicate better performance in patients. SSD=Schizophrenia Spectrum Disorder, BD=Bipolar Disorder.

**Fig. 3:**
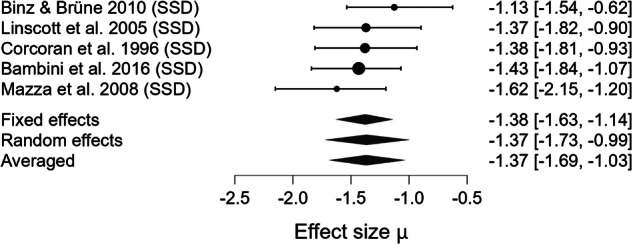
Forest plot for meta-analysis of Cooperativity impairments in mental health disorders. Bayesian Model Averaged estimates of group differences in Cooperativity impairment. Estimated (not observed) Hedges’ g values (patients >controls) are given along with 95% credible intervals in parentheses. Negative values indicate reduced Cooperativity in patients compared to controls, while positive values suggest higher level of Cooperativity in patients compared to controls. SSD=Schizophrenia Spectrum Disorder.

**Fig. 4:**
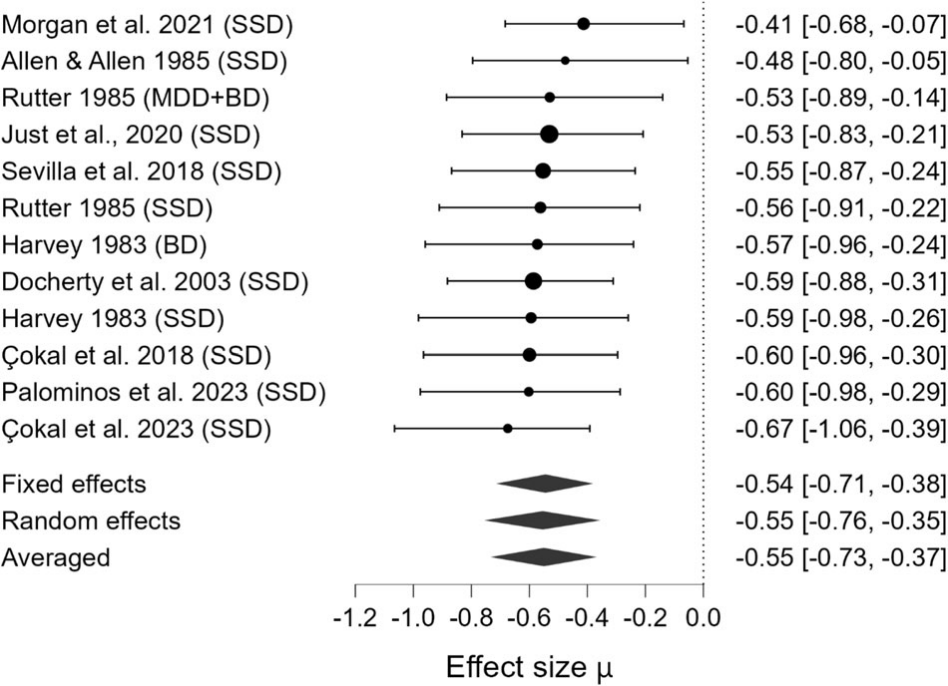
Forest plot for meta-analysis of Anaphora production impairments in mental health disorders. Bayesian Model Averaged estimates of group differences in Anaphora production impairment. Posterior estimated (not observed) Hedges’ g values (patients >controls) are given along with 95% credible intervals in parentheses. Negative values indicate that patients produced fewer or less appropriate Anaphora than controls, while positive values indicate better Anaphora production in patients relative to controls. SSD Schizophrenia Spectrum Disorder, MDD Major Depressive Disorder, BD Bipolar Disorder.

**Fig. 5:**
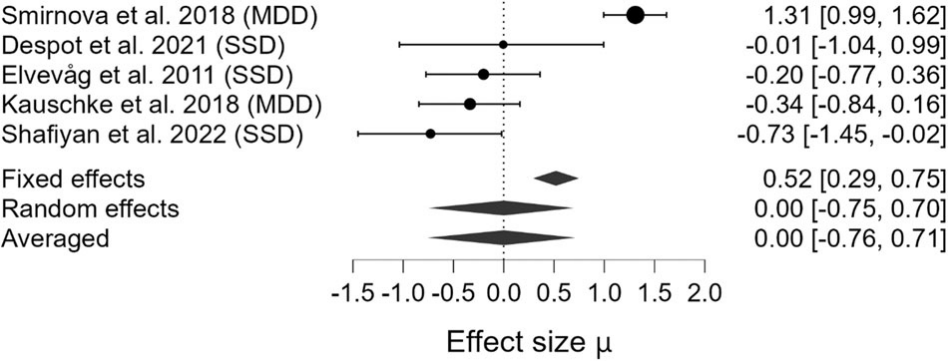
Forest plot for meta-analysis of Metaphor production impairments in mental health disorders. Bayesian Model Averaged estimates of group differences in Metaphor production impairment. Estimated (not observed) Hedges’ g values (patients >controls) are given along with 95% credible intervals in parentheses. Negative values indicate that patients produced fewer or less accurate Metaphor than controls, while positive values indicate better Metaphor production in patients relative to controls. SSD Schizophrenia Spectrum Disorder, MDD Major Depressive Disorder.

**Fig. 6:**
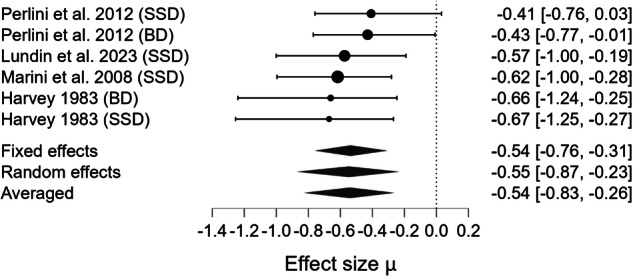
Forest plot for meta-analysis of Cohesive impairments in mental health disorders. Bayesian Model Averaged estimates of group differences in Cohesive impairment. Posterior estimated (not observed) Hedges’ g values (patients >controls) are given along with 95% credible intervals in parentheses. Negative values indicate that patients used fewer or less accurate Cohesive devices than controls, while positive values indicate better Cohesion in patients relative to controls. SSD Schizophrenia Spectrum Disorder, BD Bipolar Disorder.

**Fig. 7:**
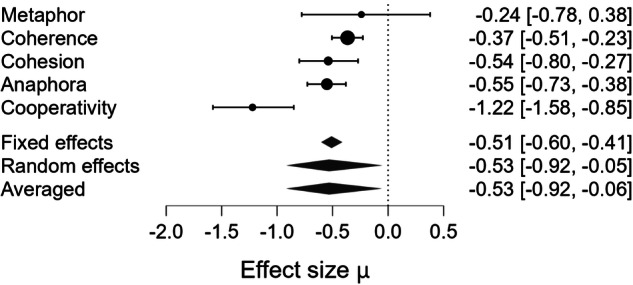
Forest plot for meta-analysis of global expressive pragmatic impairments in mental health disorders. Bayesian Model Averaged estimates of group differences in global expressive pragmatic impairments. Estimated (not observed) Hedges’ g values (patients >controls) are given along with 95% credible intervals in parentheses. Negative values indicate that patients performed worse than controls in terms of global expressive pragmatic skills, while positive values indicate better performance in patients compared to controls.

The Bayesian model-averaged meta-analyses (BMA) for each pragmatic component are summarized in Table 3, alongside estimates of heterogeneity, moderation, and publication bias. BMA provided extreme evidence for impairments in Coherence (BF₁₀ = 1118.921; Fig. 2), Cooperativity (BF₁₀ = 403.071; Fig. 3), and Anaphora (BF₁₀ = 4045.547; Fig. 4), as well as very strong evidence for impaired Cohesion (BF₁₀ = 47.616; Fig. 5). In contrast, weak evidence was found for deficits in Metaphor production (BF₁₀ = 0.358; Fig. 6). When pooling all five components, a random-effects BMA yielded moderate evidence for an overall productive pragmatic impairment (BF₁₀ = 1.930; Fig. 7).

**Table 3:** Summary of bayesian model-averaged meta-analysis of productive pragmatic impairment in mental health disorders.

Expressive Pragmatic Component	K (M)	N Patients /Controls	Hedges’g BMA [95%Crl]	BF_10_ for H_1_	Heterogenity Tau [95%Crl]	BF_rf_ for RE	Significant Moderator effect (SE)	ROBMA BF_10_ for Publication Bias and Mean Difference
**Cooperativity**	5(5)	192/147	–1.370 [–1.694, –1.028]	403.071 Extreme	0.271 [0.049, 0.724]	1.870	N/A	No publ. bias (0.800) –1.318
**Anaphora**	10(12)	326/166	–0.550 [–0.734, –0.367]	4045.547 Extreme	0.153 [0.038, 0.373]	0.820	N/A	No publ. bias (0.917) -0.446
**Cohesion**	4(6)	161/103	–0.541 [–0.828, –0.262]	47.616 Very strong	0.209 [0.042, 0.592]	0.959	N/A	Moderate publ. bias (5.163) –0.157
**Coherence**	12(16)	651/594	–0.357 [–0.513, –0.228]	1118.921 Extreme	0.168 [0.046, 0.361]	1.638	N/A	No publ. bias (0.362) –0.358
**Metaphor**	5(5)	191/150	0.0018 [–0.644, 0.718]	0.358 Weak	0.878 [0.428, 1.830]	6.379 × 10^+9^	N/A	No publ. bias (0.707) 0.058

*ROBMA* Robust Bayesian meta-analysis, *SE* Standard Error, *K* number of studies, *(M)* number of effect-size estimates, *N* sample size based on unique participant counts, *Crl* Credible Intervals, *BF*_*rf*_ *for RE* Bayes Factor for random effects over fixed effects, *BF*_*10*_ *for H*_1_ Bayes Factor for evidence for the presence of expected group differences over null hypothesis of no difference, *BMA* Bayesian Model Average, *RE* Random Effects.

Beyond the strength of evidence, the magnitude of impairment varied across pragmatic components. The largest deficit was observed for Cooperativity (observed Hedges’ g = –1.370). Moderate impairments were observed for Anaphora (observed Hedges’ g = –0.550), Cohesion (observed Hedges’ g = –0.541), and Coherence (observed Hedges’ g = –0.357). The estimated effect for Metaphor production was negligible (observed Hedges’ g = 0.002). The pooled estimate across domains (estimated Hedges’ g = –0.522) indicated a moderate overall impairment in productive pragmatic abilities. However, because only a small subset of studies assessed multiple components within the same sample, we were not able to statistically compare the magnitude of deficits across domains. Thus, although clear differences in point estimates emerged, there is currently insufficient evidence to conclude which pragmatic component is most impaired at the population level.

Between-study heterogeneity (τ) was very high for Metaphor production (τ = 0.878, CrI [0.428, 1.830], BF_rf_ = 6.379 × 10⁹), high for Cooperativity (τ = 0.271, CrI [0.049, 0.724], BF_rf_ = 1.870), and moderate for Coherence (τ = 0.168, CrI [0.046, 0.361], BF_rf_ = 1.638), Cohesion (τ = 0.209, CrI [0.042, 0.592], BF_rf_ = 0.959), and Anaphora (τ = 0.153, CrI [0.038, 0.373], BF_rf_ = 0.820). No significant moderator could be detected.

Robust Bayesian Model Averaging (ROBMA) revealed no evidence of publication bias for Anaphora production (BF₁₀ = 0.917, mean = –0.446), Cooperativity (BF₁₀ = 0.800, mean = –1.318), Coherence (BF₁₀ = 0.362, mean = –0.358), or Metaphor (BF₁₀ = 0.707, mean = 0.058). In contrast, it indicated moderate publication bias for Cohesion (BF₁₀ = 5.163, mean = –0.157).

### Studies not included in meta-analysis

K = 18^8,96,97,99–101,103,108–111,113–116,119–121^ studies for a total of 496 patients and 570 healthy controls were included in the review without meeting the M ≥ 5 requirement to be meta-analysed. Out of these, k = 16^96,97,99–101,103,107–111,113–116,121^ reported results about SSD, k = 2^110,119^ about MDD and k=1^138^reported a global score for all 3 SMIs. K = 6^96,97,106,107,109^ studies were conducted in Italian, k = 5^8,99–101,122^ in English and k = 7^110,111,113–116,119^ in another language. The pragmatic component that are explored are: Prosody (k = 5)^8,109,110,113,119^, Social Norms (k = 4)^88,96,114,115^, Turn-Taking (k = 4)^96,101,107,122^, Thematic Organisation (k = 2)^96,98^, Irony (k = 2)^96,121^, Pauses (k = 2)^99,111^, Inferences (k = 1)^98^, Implicatures (k = 1)^100^, Speech Acts (k = 1)^96^ and Global pragmatic score (k = 3)^97,108,116^. A summary of the studies’ characteristics can be found in Table 4.

**Table 4:** Summary of the studies not included in the meta-analysis.

Expressive pragmatic component	K	SSD	BD	MDD	n(pts/ctrls)	n(English/Non-English)
Prosody	5	4	1	3	143/121	1/4
Social Norms	4	4	0	0	109/95	1/3
Turn-Taking	4	4	0	0	109/203	2/2
Thematic Organisation	3	3	0	0	50/51	2/1
Irony	2	2	0	0	49/60	0/2
Pauses	2	2	0	0	62/51	1/1
Inferences	1	1	0	0	18/9	1/0
Implicatures	1	1	0	0	15/7	1/0
Speech Acts	1	1	0	0	17/28	0/1
Global	3	3	0	0	101/104	0/3
*Total*	27	26	2	3	747/793	9/19

*K* Total number of studies, n(pts/ctrls)= ratio of clinical to control participants, n(English/Non-English)= ratio of studies conducted in English to studies conducted in another language.

*SSD* Schizophrenia Spectrum Disorder, *BD* Bipolar Disorder, *MDD* Major Depressive Disorder.

Across studies of Prosody (k = 5), SSD is the most commonly studied. Acoustic analyses indicate reduced fundamental frequency (F0) range, altered stress patterns, resulting in speech that is poorly segmented and limited in its ability to convey pragmatic information^113^. In contrast, findings in affective disorders were less consistent: MDD was associated with a decreased ability to use information to mark informative structure^119^. Only results about BD, were reported grouped with SSD and MDD^8^. Thus, evidence of prosodic impairments in this group is limited. Above all, our study highlights that most studies concerned with Prosody and SMIs mostly focus on affective rather than pragmatic Prosody.

Across all four studies, SSD consistently showed marked impairments in Social Norms (k = 4) use during communication, including inappropriate conversational behavior, and violations of implicit social expectations. For instance, participants frequently failed to apply politeness and social-distance norms, tone, formality, or directness based on the social context. No results were found on MDD or BD.

Individuals with SSD show systematic disruptions in conversational Turn-Taking (k = 4), reflecting impaired coordination with interlocutors and reduced sensitivity to the temporal structure of dialog, manifesting through inconsistent turn initiation and reduced responsiveness^96^ or aberrant turns, including overly short, fragmented, or error-filled contributions^103^. Interaction-based studies^101,107^ demonstrate that these difficulties extend to real conversational settings, where SSD speakers show atypical timing, misaligned responses, and difficulty maintaining reciprocal conversational flow. No results were found on MDD or BD.

Across all studies, SSD shows robust impairments in Thematic Organization (k = 3). Narratives are often produced with fragmented global themes, abrupt topic shifts, and poorly integrated story elements^98^. Difficulties sustaining a central message, with narratives displaying loose or poorly articulated thematic links were also observed^96^. These behavioral findings are reinforced by computational evidence: SSD patients exhibit poorer “on-topic” performance compared to controls, indicating unstable thematic maintenance during structured picture-description and storytelling tasks^75^. Taken together, these studies suggest that SSD speakers struggle to maintain an overarching theme and to integrate narrative components into a unified whole. No evidence was found for MDD or BD.

Irony (k = 2) emerged as the most challenging expressive task for SSD patients compared to direct, indirect, and deceitful communicative acts^96^. These difficulties cannot be fully explained by Theory of Mind or Executive functions deficits, suggesting a more fundamental impairment in pragmatic inferential processing underlying Irony^106^. No study were found on MDD or BD.

Across studies examining Pauses (k = 2), SSD patients consistently show abnormalities in temporal speech patterns, particularly in relation to contextual processing. For instance, thought-disordered SSD patients lose the normal sensitivity to contextual predictability, producing similar pause rates before both predictable and unpredictable words, whereas healthy controls and non-thought-disordered SSD patients pause more before unpredictable lexical items^111^. This reflects a disruption in integrating contextual demands and aligns with broader contextual insensitivity in SSD. It is further reported that SSD speakers exhibit altered pausing profiles during speech production, with atypical timing and distribution of silent pauses contributing to reduced fluency and altered syntactic structures^99^. No studies were found examining pauses in MDD or BD.

Across the only available study on Inferences (k = 1), individuals with SSD produced fewer inferential ideas when describing visual scenes, indicating reduced ability to derive implied information rather than relying solely on what is explicitly shown^98^. Such reduced inferencing reflects a shift toward concrete thinking, limiting patients’ capacity to elaborate meaningfully beyond surface-level cues. No results were found for MDD or BD.

The only study (k = 1) reporting results on Implicatures, revealed that patients produced significantly lower connector words triggering implicatures in context than controls. These differences persisted even after controlling for verbal IQ, suggesting a core pragmatic impairment rather than a generalized verbal deficit^100^. No studies were found on MDD and BD.

Evidence on the production of Speech Acts (k = 1) shows that individuals with SSD demonstrate clear impairments in producing appropriate direct and indirect communicative acts, frequently failing to generate responses that were pragmatically coherent with the conversational context. Patients often produced overly literal utterances, indicating difficulties generating requests, assertions, or clarifications appropriate in context^96^ further supporting reduced sensibility to contextual demands. No results were found for MDD or BD.

Finally, across studies assessing Global Pragmatic Abilities (k = 3), SSD consistently show marked global impairments, spanning multiple communicative behaviors such as managing conversational relevance, producing context-appropriate responses, and coherently organizing their speech. Those impairments linked only modestly to cognitive or Theory-of-Mind components^108^. Taken together, these evidences are consistent with the overall results presented in this study. Finally, no study reported Global scores for MDD or BD.

Across all productive pragmatic domains reviewed, SSD showed the most pervasive and consistent impairments, affecting multiple dimensions of pragmatics These deficits reflect a broad disruption in the ability to generate context-appropriate, coherent, and socially coordinated communicative behavior, extending well beyond any single pragmatic skill. Although more limited and less significant in magnitude, some evidence of impairments were also found in MDD and BD, especially regarding Prosody. However, evidence for MDD and BD was scarce or absent in most domains, underscoring a clear gap in the literature.

## Discussion

To our knowledge, this is the first study to provide a systematic qualitative and quantitative review of expressive pragmatic impairments in SMIs. Our literature search identified five core areas of pragmatic impairment with a sufficient number of studies for a quantitative synthesis: Coherence, Cooperativity, Cohesion, Anaphora, and Metaphor. 10 other domains including Social Norms, Turn-Taking were also identified as features of expressive pragmatic impairment studied in people with SMIs. Of the three SMIs investigated, SSD was the most commonly studied, followed by MDD and BD. We observed a moderate degree of Global pragmatic impairment in SMIs, with strong evidence for impairments in Coherence, Cooperativity, Anaphora, and Cohesion but only weak evidence for Metaphor production.

To our knowledge, this is the first work to systematically qualify and quantify expressive pragmatic impairments in SMIs. Other studies have either focused on comprehension^11^ or on specific sub-domains of pragmatics, such as coherence. Moreover, this study is the first to focus on various SMIs, whereas most expressive pragmatic studies focus on one specific disorder (SSD in most cases). As a result, this study is the first to provide an overview of pragmatic deficits across multiple SMIs, thus highlighting transdiagnostic patterns of impairments. Those patterns have multiple implications: Although data on variations in the severity of impairments across diagnosis is limited, our pooled results do indicate the presence of more pronounced deficits in SSD as opposed to MDD/BD. Those observations are consistent with the degree of functional deficits known to separate these illnesses. If confirmed by further studies, this makes pragmatic abilities a prime target for neuroimaging inquiries of varying functional burden of SMIs: For instance, future work could explore whether the same neuronal pattern responsible for pragmatic abilities differ in magnitude across diagnosis supporting a transdiagnostic conceptualization of SMIs. Finally, our study also suggests that pragmatic measurement/remediation tools such as AbaCo and PragmaCom could be extended beyond SSD and be beneficial for other SMIs.

In addition, this study uncovered the current lack of consensus regarding which expressive pragmatic features are clinically relevant: Although many studies focus on coherence, a plethora of other expressive pragmatic features are investigated. As a result, this study provides a very broad overview of potential pragmatic impairments in SMIs, given the extensive literature search conducted to scope potential expressive pragmatic impairments in SMIs. Thus, our search is more granular than previous work in the field^37^, as we cover unique search terms like Coherence, Cooperativity, Anaphora, etc., which were often omitted in previous works.

Finally, this study also covers a large temporal period, spanning over ≈40 years of work (as opposed to ≈25 years in previous works^37^).

One key limitation of our study is the overrepresentation of SSD compared to MDD and BD, which implies that our findings primarily reflect deficits in SSD. Thus, overall evidence of productive pragmatic impairments in SMIs should be interpreted with caution. Although the data reported on Metaphor are more balanced in terms of SMIs diagnostics (2 out of 5 studies report data on MDD and 3 on SSD), this poses another challenge: One study^95^ reported data contradicting the other studies, namely that MDD participants produced more metaphors than controls. Therefore, the results of the Metaphor BMA are biased towards null.

Another significant limitation arises from the heterogeneity in expressive pragmatic measurement used in the included studies: (i) Similar pragmatic metrics were quantified through different methods such as manual^72,117^ versus automated^75^ (computational) measures for Coherence, Anaphora and Cohesion; (ii) Various tasks were used to elicit speech data across studies. As a result, the same pragmatic phenomenon was sometimes assessed on different types of data. For instance, some studies measured Coherence in picture description tasks^69^, whereas others relied on free speech samples^77^; (iii) Some studies only reported general pragmatic scores that could not be broken down into finer components^116^. Taken together, this heterogeneity limits the comparability of the studies’ results.

Most included studies only include “healthy-enough” patients whose symptoms do not prevent them from participating in a research study or from providing informed consent (mainly outpatients). Therefore, the data reported might not be generalizable to the whole populations as results might be biased toward healthier participants. In particular, this bias could explain why we did not detect an effect of symptom severity on pragmatic abilities. Therefore, it should not be inferred that such an effect is not present in the population. Moreover, this lack of effect could also be explained by the relatively small sample size of included studies which significantly reduce statistical power to detect such an effect (type II error). However, if empirically verified, such a lack of effect could be indicative of pragmatic difficulties present in SDD (and SMIs), irrespective of state-like variations. This interpretation would be consistent with recent evidence suggesting that pragmatic impairment reflects a deeper, partially state-independent vulnerability linked to underlying social-cognitive and executive mechanisms, rather than transient consequences of acute psychopathology^37^.

Finally, we noted that the term “Referential Noun Phrases” was sometimes used in the literature to capture anaphoric phenomenon. While our initial search string (Supplementary Table S1) did not include this term, we performed an a posteriori search for those articles, which yielded k = 5 studies^104,105,117,118,123^, four^104,105,117,118^ of which were subsequently included in the review and meta-analysis during the revision process. One^123^ was published more recently and thus couldn’t be included in this study. However, we do acknowledge that due to variations in terms used, our review of Anaphora production might be less comprehensive than the other pragmatic components investigated.

Given the strength of evidence for impairments in SMIs—most particularly in SSD— as opposed to controls, our results advocate for the use of expressive pragmatic aspects of language as a behavioral marker when assessing SMIs. Pragmatic deficits could potentially be incorporated into digital mental health tools aimed at SMIs’ diagnosis, thus offering a cheaper and more inclusive alternative to mainstream mental health care^124^. However, it also uncovered the current lack of consensus on clinically relevant features. Thus, this study should be used as a preliminary step in identifying such features as it highlighted the high degree of impairments in multiple pragmatic sub-domains.

Our work also advocates for the assessment and treatment of expressive impairments to be incorporated as part of a holistic treatment plan for SMIs. In addition, given the intrinsic relationship between pragmatic impairments and socio-occupational functioning^18,20^ special attention should be put on pragmatic remediation. In this respect, interventions such as social skills group training or the Pragmatics of Communication (PragmaCom) show promising results in restoring pragmatic skills both in SSD^125^ and Autism Spectrum Disorder^126^. Following proper expressive pragmatic impairments remediation, we can hypothesize a positive increase in socio-occupational functioning.

From a methodological point of view, our study also highlights the lack of a standardized approach to measuring expressive pragmatic skills in both patients and controls. Although some standardized tools exist (e.g., AbaCo and APACS for Italian speakers), there is a considerable variability in which components of pragmatics are assessed and by which means. Although Coherence and Cohesion can be measured computationally^85^, Cooperativity, Anaphora, and Metaphors are mainly quantified manually. Thus, future studies should focus on designing standardized tools to systematically and computationally assess expressive pragmatic production impairments in clinical populations to potentially include them in clinical practice. To this aim, Large-Language Models (LLM) offer a promising avenue as preliminary studies report high accuracy between LLMs and human ratings (e.g., formal thought disorder) following supervised training (labeled data)^127^. Developing such automated ratings based on human-rated measures of pragmatics would allow for quick, systematic and non-invasive evaluation of patients’ pragmatic abilities.

Another key finding of this study is that Cohesion and Anaphora showed larger and more robust impairments than Coherence. This suggests that pragmatic impairments in SSD and SMIs may be more pronounced at more focused levels of discourse organization, such as referential expressions and textual connectivity, than at the broader level of coherence. Consequently, approaches focusing predominantly on coherence may underestimate deficits, especially since coherence can be partially preserved despite disruptions in more precise pragmatic mechanisms such as anaphora production and cohesion. This further highlight the need for systematic and quantifiable measures of pragmatic measurements in clinical settings.

Regarding moderating effects, we considered including speech elicatation tasks in the moderator analysis. However, given the heterogeneity and imbalance of elictation tasks used by the meta-analyzed studies, we decided against it since sufficient data would not have been available to conduct a reliable analysis. Thus, the generalizability and interpretability of our results would have been limited. Nevertheless, we believe that such analysis would be highly relevant to explore task-specific effects and to determine which tasks are best suited to analyze productive pragmatic impairments in SMIs. Therefore, our study also calls for future work to focus on such questions.

Finally, our review highlighted the clear lack of studies focusing on MDD and BD, although the existing studies show significant proof of impairments. More studies are thus needed to better qualify and quantify expressive pragmatic deficits in those SMIs, especially regarding Cooperativity as none were found on either MDD or BD. This study also revealed that most of the literature is diagnostic-specific. We recommend adopting a transdiagnostic approach to qualify and quantify expressive pragmatic deficits across diagnoses. This approach is crucial for informing both theoretical models of language dysfunction in psychiatry and the development of targeted, diagnosis-neutral interventions. Finally, this work demonstrates that further work is needed, especially in Cooperativity and Cohesion where the number of available studies is limited despite significant evidence of impairments. For a summary of the research gap highlighted by this study and the implications/recommendations they warrant, see Table 5.

**Table 5:** Summary of research gap and implications/recommendations.

Research gaps	Implications/recommendations
Lack of consensus on which expressive pragmatic component are clinically relevant.	Define which expressive pragmatic features are clinically relevant.
Lack of standardized tools for measuring broad range of expressive pragmatic impairments.	Develop standardized tools for corpus linguistic studies systematically in clinical populations.
Automated approaches exist for Coherence, Anaphora, and Cohesion but not for Cooperativity and Metaphor.	Test and implement automated approaches to measure Cooperativity and Metaphor use.
Limited research on expressive pragmatic impairments in MDD and BD.	Conduct more studies to qualify and quantify deficits in these conditions, especially in Cooperativity.
Diagnosis-specific framework employed in most studies	Adopting a transdiagnostic approach to qualify and quantify expressive pragmatic impairments across SMIs.
Focus has been predominantly on Coherence and Anaphora impairments.	Expand research to include Cooperativity and Cohesion.

## Supplementary information

Supplementary Material (revised)

## Data Availability

No datasets were generated or analysed during the current study.
