# Supplementary material for: Expressive pragmatic language in mood and psychotic disorders: a systematic review and meta-analysis
Source: Schizophrenia (Heidelb). 2026 Feb 14;12(1):31. doi: 10.1038/s41537-026-00733-2 (PMC13022284; doi:10.1038/s41537-026-00733-2)
Supplement: Supplementary file 1 — Supplementary Material (revised) [file 41537_2026_733_MOESM1_ESM.docx]

**Supplementary Material:**

**S1. Categorization of Studies Pragmatic Components**

Given the heterogeneity of definitions of pragmatic components, we acknowledge that the distinction between some of the components included in this study might be unclear. In particular, the notions of coherence, cohesion, Anaphora, and deixis might seem to overlap. Therefore, we will (i) clarify the definitions used in this study for these 4 components and show how they differ from each other (ii) provide the systematic decision process used to categorize the included studies. However, it should be noted that the categorization described below is debated and is not the only accepted ones. Therefore, the reader should keep in mind that the categorization used in this work partially result from theoretical choice.

**S1.1. Definitions**:

**Coherence**: Following the linguistic definition^1,2^ that has been operationalized in psychiatry^3^, we defined coherence as the “logical consistency -- i.e., physical, motivational, and psychological continuity and causal linkage -- between the events, actions, and states described in individual clauses“. In other words, coherence is the overall logical flow and unity of a text/discourse. To achieve a coherent discourse, the speaker can use (i) implicit linguistic devices such as rhetorical relations^4^ (e.g. John went to bed. **Ø** He was tired); (ii) explicit linguistic devices, known as cohesive markers (e.g. John went to bed **because** he was tired).

In line with this definition, measures of semantic similarity between clauses or sentences, often derived from computational (NLP) methods, are considered operational proxies for assessing aspects of coherence, specifically the strength of semantic relationships within the discourse. Thus, in accordance with previous work in psychiatry^10,11^, outcomes of semantic similarity measures were categorized under ‘’coherence’’, as this is the closest pragmatic sub-category for which semantic similarity was used as a proxy by authors.

**Cohesion**: In this work, we define cohesion as the set of explicit surface-level linguistic devices that can be used to signal relations within a text and thereby contribute to its coherence^1^. Cohesive devices can be of multiple nature^2^: referential expressions (e.g., pronouns, demonstratives), substitutions, ellipsis, conjunctions, and lexical substitution. Based on this definition, it is to note that coherence and cohesion are two separate but complementary concepts: cohesion is a means amongst other to achieve coherence. In addition, cohesive devices have to be separated from referential expressions, such as Anaphora, as their interpretation.

**Anaphora**: In this work, operationalized Anaphora as ‘’a relation between two or more linguistic expressions, wherein the interpretation of one (called an anaphoric expression) [expressed through a noun phrase^5^] is in some way determined by the interpretation of the other (called an antecedent)’’^6^. A common example of anaphoric expression is Definite Noun Phrases such as pronouns pointing to a referent introduced earlier (or after in the case of cataphoras) in the discourse (e.g., **John** was hungry. **He** ate a sandwich) or 3^rd^ Person Definite Noun Phrases (I saw **my supervisor** today. **The man** was happy with my progress)^5^. However, other anaphoric expressions do exist such as but not restricted to Zero Anaphora (e.g. John’s **keys** are on the table. Mary’s [**Ø keys**] too)^7^ and Bridging-Cross Reference Anaphora (John walked into **the library**. The music reading room [**Ø of the library**] had just been refurbished)^6^. In this definition, the importance of the linguistic nature of the antecedent must be stressed (as opposed to the context of utterance for deixis^5^).

**Deixis**: In this work, deixis was operationalized as expressions whose interpretation depends on the context of utterance (e.g. ‘’that’’, ‘’yesterday’’, ‘’you’’)^6^. In other words, the dexis interpretation depends on extra-linguistic knowledge as opposed to the direct linguistic context The critical distinction from anaphora is the locus of the reference: anaphora relies on an intra-textual antecedent, while deixis relies on the extra-linguistic context of the utterance

**S1.2. Categorization Process**: Although, Coherence, Cohesion, Anaphora, and Deixis are separate concepts, there is significant overlap between them through a hierarchical relationship: Coherence can be achieved through cohesion, cohesion can be achieved through anaphora and deixis. We decided against categorizing all these concepts under the umbrella of coherence for the following reasons: (i) Studies report results with various granularity (e.g., some report general results for coherence, while others report specific results regarding Anaphora). Therefore, we felt that such simplification would have obscured important theoretical and empirical distinctions across levels of discourse organization. (ii) There is significant evidence that both Anaphora^8^ and deixis^9^ show significant impairments in SMIs, particularly psychosis. Thus, maintaining a finer distinction between the different levels of coherence enables us to more precisely capture the nature and locus of pragmatic disruptions across studies.

As a result, we categorized studies following a top-down decision process starting with Coherence, based on the amount of detail provided by the study (see Figure S1):

**Coherence**:

- The study reports quantifiable result on either/or the overall logical flow or unity of participant's discourse.
- The study does not report results on explicit linguistic devices used to achieve coherence (else → Cohesion)

**Cohesion**:

- The study reports quantifiable results on cohesive devices used to achieve coherence.
- The cohesive devices reported are not exclusively referential in nature (else → Anaphora or Deixis).

**Anaphora**:

- The study reports quantifiable results on anaphoric expressions (i.e. Interpretation depends on the linguistic context).
- The study does not exclusively report results on deictic expressions (i.e. Interpretation depends on the extra-linguistic context) (else → Deixis)

**Deixis**:

- The study reports quantifiable results on deictic expressions (i.e. Interpretation depends on the extra-linguistic context).
- The study does not report results on anaphoric expressions (i.e. Interpretation depends on the linguistic context) (else → Anaphora) or other cohesive devices (else → cohesion)

**S1.4. Note on Thematic Organization as Distinct Pragmatic Feature**: Although conceptually related, in this work, we regarded thematic organization as separate from coherence for the following reason: Thematic Organization refers to one’s topic management in discourse, such as how/when topics are introduced, maintained, shifted, etc.^12^ However, it is distinct from coherence and lower-level components such as cohesion as it is not concerned with the discourse unity (i.e., the formal (explicit/implicit) linguistic devices used to ensure the logical continuity of discourse. To illustrate the independence of these two pragmatic phenomena, please consider the imaginary discourse excerpt in (1), which features appropriate thematic organization (the topic is maintained) but poor coherence (logical continuity is hard to follow):

(1) **Topic: “My job at the grocery store”**

*“I work at the grocery store. The vegetables know when you’re tired. Customers sometimes look like Thursday. The cash register is fine, but the air smells too purple when it rains.”*

In addition, the opposite can be observed in patients whose speech feature tangentiality, as they might provide a coherent but off-topic answer to a question^13^.

**Figure S1: Top-Down Categorization Process for Coherence, Cohesion, Anaphora and Deixis Studies**


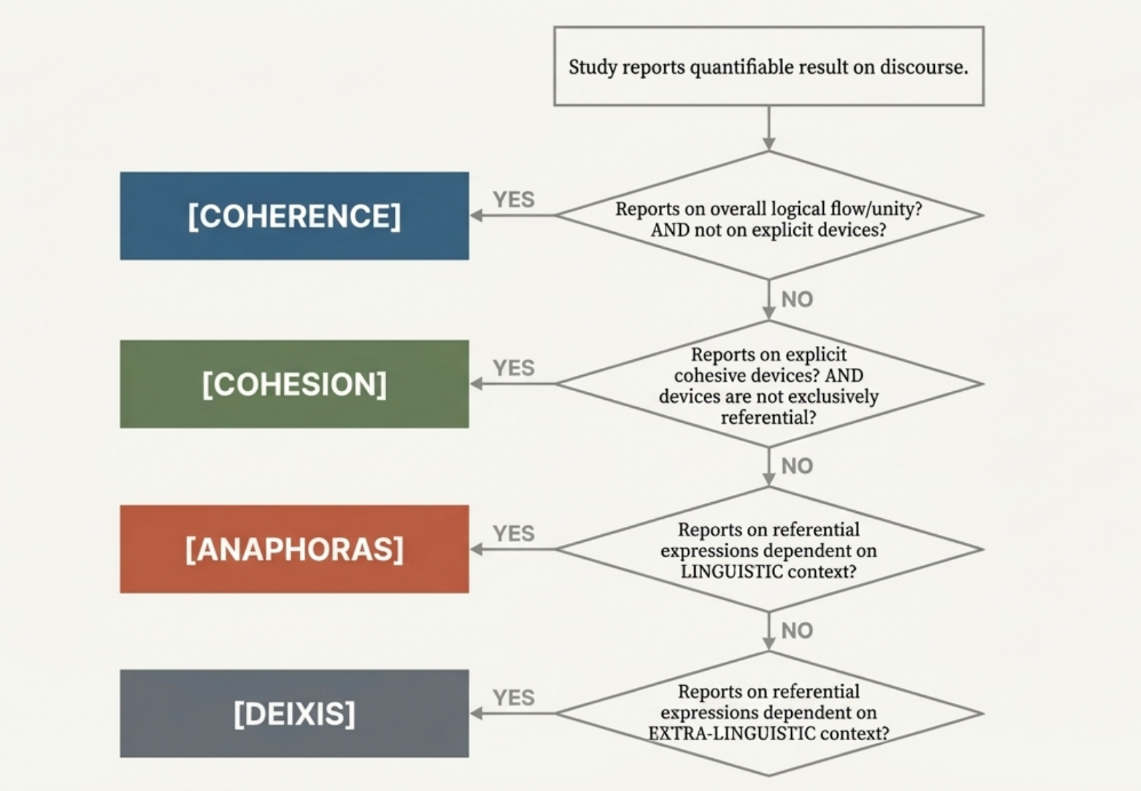


**Table S1: Search Strings**

| PubMed | Search: (SSD [Title/Abstract] OR seasonal affective [Title/Abstract] OR dysthymi* [Title/Abstract] OR depress* [Title/Abstract] OR delusion* [Title/Abstract] OR BD [Title/Abstract] OR MDD [Title/Abstract] OR bipolar* [Title/Abstract] OR mania* [Title/Abstract] OR manic [Title/Abstract] OR mood disorder* [Title/Abstract] OR psychoses [Title/Abstract] OR psychosi* [Title/Abstract] OR psychoti* [Title/Abstract] OR schizo* [Title/Abstract] ) AND ((anaphor* [Title/Abstract] OR deix* [Title/Abstract] OR explicature* [Title/Abstract] OR figurative language* [Title/Abstract] OR Grice* [Title/Abstract] OR idiom* [Title/Abstract] OR implicature* [Title/Abstract] OR implicit meaning* [Title/Abstract] OR indirect request* [Title/Abstract] OR intonation* [Title/Abstract] OR ironi* [Title/Abstract] OR irony [Title/Abstract] OR maxims of conversation [Title/Abstract] OR metaphor* [Title/Abstract] OR non literal [Title/Abstract] OR prosod* [Title/Abstract] OR proverb* [Title/Abstract] OR speech act* [Title/Abstract] OR topic model* [Title/Abstract] OR turn tak* [Title/Abstract] ) OR ((language* [Title/Abstract] OR speech* [Title/Abstract] OR linguistic* [Title/Abstract] ) AND (coherence* [Title/Abstract] OR discourse* [Title/Abstract] OR inferen* [Title/Abstract] OR Interrupt* [Title/Abstract] OR paus* [Title/Abstract] OR paus* [Title/Abstract] OR social norm* [Title/Abstract] OR on topic* [Title/Abstract] OR presuppos* [Title/Abstract] OR humor* [Title/Abstract] OR humour* [Title/Abstract] OR pragmatic* [Title/Abstract] OR cohesi* [Title/Abstract] OR disconnect* [Title/Abstract]))) Filters: English |
| --- | --- |
| Scopus | ( TITLE-ABS ( bipolar* OR "seasonal affective" OR dysthymi* OR delusion* OR mania* OR manic OR mdd OR depress* OR "mood disorder*" OR psychoses OR psychosi* OR psychoti* OR schizo* OR ssd OR bd ) ) AND ( TITLE-ABS ( anaphor* OR deix* OR explicature* OR "figurative language*" OR grice* OR idiom* OR implicature* OR "implicit meaning*" OR "indirect request*" OR intonation* OR ironi* OR irony OR "maxims of conversation" OR metaphor* OR "non literal" OR prosod* OR proverb* OR "speech act*" OR "topic model*" OR "turn tak*" ) OR ( TITLE-ABS ( language* OR speech* OR linguistic* ) AND ( TITLE-ABS ( coherence* OR inferen* OR interrupt* OR paus* OR "off topic*" OR "on topic*" OR "social norm*" OR presuppos* OR pragmatic* OR cohesi* OR disconnect* AND discourse* OR humor* OR humour* ) ) ) ) AND ( LIMIT-TO ( DOCTYPE,"ar" ) ) AND ( LIMIT-TO ( LANGUAGE,"English" ) ) |

Notes: * indicates a truncation.

**Table S2: Inclusion/Exclusion Criteria (Disorders)**

| **Inclusions** | **Exclusions** |
| --- | --- |
| Schizophrenia, Brief Psychotic Disorder, Delusional Disorder, First Episode Psychosis, Postpartum Psychosis, Schizoaffective, Schizophreniform | Schizotyped/Schizotypy |
| Major Depressive Disorder, Depression, Postpartum Depression, Seasonal Affective Disorder, dysthymia |  |
| Bipolar Disorder, Manic Depression, |  |

**Table S3: Key Variables and Moderators for the Case-Control Comparisons in the Meta-Analysis**

| **Study**^[[1]](#footnote-1)^ | **Expressive Pragmatic Component**^[[2]](#footnote-2)^ | | **Diagnsotic** | | **n(pts/ctrls)** | **Mean(SD_pts_)** | | **Mean(SD_CTRLS_)** | **Effect Size (g)** | | **Standard Error (SE)** | | **Mean age pts** | | **% males pts** | | | **Symptom severity** | **English** | | | **Quality Score** |
| --- | --- | --- | --- | --- | --- | --- | --- | --- | --- | --- | --- | --- | --- | --- | --- | --- | --- | --- | --- | --- | --- | --- |
| Allen & Allen 1985^*^ | Anaphora (Omitted Referents) | SSD | | 19/9 | | 7.936 (13.824) | 8.04 (13.18) | | | -0.007 | | 0.398 | | 51.16 | | NA | N/A | | | Yes | 9 | |
| Allen & Allen 1985^*^ | Coherence (Connections per Idea) | SSD | | 19/9 | | 1.661 (0.645) | 1.74 (0.36) | | | -0.132 | | 0.398 | | 51.16 | | NA | N/A | | | Yes | 9 | |
| Alonso-Sanchez 2022 | Coherence (Average Similarity of Words in Full picture description) | SSD | | 46/36 | | 0.337 (0.02) | 0.332 (0.02) | | | -0.247 | | 0.223 | | 22 | | 0.77 | 0.367 | | | Yes | 13 | |
| Bambini et al. 2016 | Cooperativity (Interview^[[3]](#footnote-3)^) | SSD | | 47/35 | | 39.72 (3.21) | 43.6 (1.17) | | | -1.5068 | | 0.2505 | | 39.74 | | 0.62 | 0.263 | | | No | 12 | |
| BenMoshe et al. 2024 | Coherence (Derailment^[[4]](#footnote-4)^) | SSD | | 23/25 | | 0.225 (0.016) | 0.225 (0.016) | | | 0 | | 0.289 | | 25.46 | | 1 | 0.318 | | | No | 12 | |
| Binz & Brüne 2010 | Cooperativity (Grice’s Maxims pooled) | SSD | | 49/29 | | 1.955 (1.719) | 0.705 (0.887) | | | -0.843 | | 0.243 | | 37.3 | | 0.49 | 0.238 | | | No | 8 | |
| Çokal et al. 2018 | Anaphora (pooled) | SSD | | 30/15 | | 0.29(0.25) | 0.11(0.13) | | | -0.79 | | 0.32 | | 44 | | 0.76 | 0.283 | | | Yes | 13 | |
| Çokal et al. 2023 | Anaphora (pooled) | SSD | | 31/27 | | 5.34 (5.92) | 0.33 (0.78) | | | -1.01 | | 0.25 | | 36.48 | | 0.58 | 0.283 | | | No | 12 | |
| Corcoran et al. 1996^*^ | Cooperativity (Grice’s Maxims Total) | SSD | | 38/13 | | 15.24(2.59) | 18.54(1.1) | | | -1.40 | | 0.35 | | 33.37 | | N/A | N/A | | | Yes | 9 | |
| Despot et al. 2021 | Metaphors (Controlled and semi-controlled pooled) | SSD | | 5/5 | | 0.056 (0.509) | 0.061 (0.529) | | | −0.0087 | | 0.632 | | N/A | | N/A | N/A | | | No | 6 | |
| Docherty et al. 2003 | Anaphora (pooled) | SSD | | 48/28 | | 0.40 (0.57) | 0.125 (0.132) | | | -0.67 | | 0.24 | | 36 | | 0.54 | 0.48 | | | Yes | 13 | |
| Elvevåg et al. 2011 | Metaphors | SSD | | 21/21 | | 0.80 (1.5) | 1.23 (2.0) | | | -0.238 | | 0.309 | | 33.52 | | NA | N/A | | | No | 8 | |
| Figueroa-Barra et al. 2022^*^ | Coherence (Mean cosine similarity between question-answer) | SSD | | 49/84 | | 0.767(0.025) | 0.79 (0.021) | | | -0.916 | | 0.19 | | 27.11 | | 0.6 | 0.566 | | | No | 9 | |
| Gargano et al. 2022 | Coherence (Global) | SSD | | 133/133 | | 0.06 (0.47) | 0.0 (0.0) | | | -0.18 | | 0.122 | | 28.93 | | 0.6 | 0.02 | | | No | 7 | |
| Harvey 1983^*^ | Anaphora (Unclear References and  Ambiguous References pooled) | SSD | | 20/10 | | 0.075 (0.105) | 0.000 (0.014) | | | -0.84 | | 0.402 | | 32.6 | | 0.2 | N/A | | | Yes | 13 | |
| Harvey 1983* | Anaphora (Idem) | BD | | 20/10 | | 0.058 (0.100) | 0.000 (0.014) | | | -0.681 | | 0.397 | | 30.15 | | 0.3 | N/A | | | Yes | 13 | |
| Harvey 1983* | Cohesion(Total Cohesion) | SSD | | 20/10 | | 1.52 (0.485) | 2.08 (0.47) | | | -1.134 | | 0.414 | | 32.6 | | 0.2 | N/A | | | Yes | 13 | |
| Harvey 1983* | Cohesion (Idem) | BD | | 20/10 | | 1.619 (0.386) | 2.08 (0.47) | | | -1.082 | | 0.411 | | 30.15 | | 0.3 | N/A | | | Yes | 13 | |
| Hoffman et al. 1986 | Coherence (Total Deviance) | SSD | | 39/40 | | 6.26 (8.95) | 1.57 (3.69) | | | -0.6816 | | 0.231 | | 26.6 | | NA | N/A | | | Yes | 11 | |
| Hoffman et al. 1986 | Coherence (Idem) | BD | | 24/40 | | 12.29 (17.40) | 1.57 (3.69) | | | -0.963 | | 0.271 | | 34.7 | | NA | N/A | | | Yes | 11 | |
| Just et al. 2020^*^ | Coherence(automatically derived coherence metrics) | SSD | | 40/20 | | 0.2713(0.07)^[[5]](#footnote-5)^ | 0.29(0.05)^[[6]](#footnote-6)^ | | | −0.332 | | 0.27 | | 43.8 | | 0.55 | 0.224 | | | No | 12 | |
| Just et al., 2020^*^ | Anaphora (Ambiguous Referents) | SSD | | 40/20 | | 0.11(0.21)^[[7]](#footnote-7)^ | 0.0145(0.0481)^[[8]](#footnote-8)^ | | | -0.574 | | 0.27 | | 43.8 | | 0.55 | 0.224 | | | No | 12 | |
| Kauschke et al. 2018 | Metaphors | MDD | | 26/32 | | 14.98 (9.79) | 19.83 (14.29) | | | -0.383 | | 0.266 | | 37.26 | | 0.38 | N/A | | | No | 12 | |
| Linscott 2005 | Cooperativity (Grice’s Maxims) | SSD | | 20/26 | | 10.8 (3.9) | 7.2 (0.3) | | | -1.3753 | | 0.33 | | 30.5 | | 0.8 | N/A | | | Yes | 12 | |
| Lundin et al. 2023 | Cohesion (Deep Cohesion) | SSD | | 32/15 | | 0.53 (0.85) | 1.05 (0.67) | | | -0.6404 | | 0.319 | | 41.66 | | 0.53 | 0.192 | | | Yes | 10 | |
| Marini et al. 2008 | Coherence (Global Coherence) | SSD | | 29/48 | | 13.667 (13.787) | 0.533 (2.281) | | | -1.509 | | 0.26 | | 28.93 | | 0.6015 | 0.123 | | | No | 8 | |
| Mazza et al. 2008 | Cooperativity (Grice’s Maxims pooled) | SSD | | 38/44 | | 1.672(1.143) | 0.146(0.231) | | | -1.95 | | 0.27 | | 38.5 | | 0.79 | 0.143 | | | No | 8 | |
| Morgan et al. 2021 | Anaphora (Ambiguous Pronouns pooled) | SSD | | 16/13 | | N/A | N/A | | | -0.164 | | 0.19 | | 24.5 | | 0.81 | N/A | | | Yes | 12 | |
| Morgan et al. 2021 | Coherence (pooled) | SSD | | 16/13 | | N/A | N/A | | | -0.59 | | 0.2 | | 24.5 | | 0.813 | N/A | | | Yes | 12 | |
| Palominos et al., 2023 | Anaphora (explicit referential re-use) | SSD | | 20/20 | | 0.052 (0.021) | 0.036 (0.018) | | | -0.801 | | 0.328 | | 35.7 | | 0.6 | 0.593 | | | No | 9 | |
| Parola et al. 2023 CH | Coherence (Second-order Coherence) | SSD ch | | 51/42 | | N/A | N/A | | | -0.2 | | 0.199 | | 27.2 | | 0.78 | 0.254 | | | No | 14 | |
| Parola et al. 2023 DK | Coherence (Idem) | SSD dk | | 111/129 | | N/A | N/A | | | -0.2 | | 0.135 | | 26.9 | | 0.78 | 0.068 | | | No | 14 | |
| Parola et al. 2023 GE | Coherence (Idem) | SSD ge | | 25/29 | | N/A | N/A | | | -0.26 | | 0.284 | | 29.2 | | 0.78 | 0.127 | | | No | 14 | |
| Perlini et al. 2012 | Coherence (Global Coherence) | SSD | | 30/30 | | 9.28 (8.26) | 4.16 (6.00) | | | -0.7 | | 0.265 | | 39.7 | | 0.8 | N/A | | | No | 13 | |
| Perlini et al. 2012 | Coherence (Idem) | BD | | 30/30 | | 7.90 (7.34) | 4.16 (6.00) | | | -0.5507 | | 0.263 | | 44.83 | | 0.37 | N/A | | | No | 13 | |
| Rutter 1985 | Anaphora (Ambiguous Phoric References) | SSD | | 35/10 | | 6.8 (9.7) | 1.5 (2.3) | | | -0.599 | | 0.364 | | NA | | NA | N/A | | | Yes | 5 | |
| Rutter 1985 | Anaphora (Idem) | MDD+BD | | 7/10 | | 3.8 (7.0) | 1.5 (2.3) | | | -0.45 | | 0.49 | | NA | | NA | N/A | | | Yes | 5 | |
| Sevilla et al. 2018 | Anaphora (pooled) | SSD | | 40/14 | | 1.31(2.38) | 0.30(0.86) | | | -0.54 | | 0.31 | | 41.28 | | 0.6 | 0.255 | | | No | 14 | |
| Shafiyan et al. 2022 | Metaphors | SSD | | 15/15 | | 1.00 (1.253) | 3.333 (3.244) | | | -0.9232 | | 0.384 | | 47.8 | | 0 | N/A | | | No | 11 | |
| Smirnova et al. 2018 | Metaphors | MDD | | 124/77 | | 2.55 (0.84) | 1.40 (0.83) | | | 1.3701 | | 0.1599 | | 42 | | 0.24 | N/A | | | No | 12 | |
| Tagamets et al., 2014 | Coherence (Personal Thematic Coherence) | SSD | | 11/11 | | 0.817 (.030) | 0.848 (.043) | | | -0.8044 | | 0.443 | | 40 | | 0.82 | N/A | | | Yes | 12 | |

NOTES: n(pts/ctrls)= ratio of clinical to control participants, Mean(SD_pts_)= Mean and standard deviation for patient population, Mean(SD_CTRLS_)= Mean and standard deviation for control population, g= Hedges’g, SE=Standard Error.

1. Cohesion and Coherence: Linguistic Approaches. 591–595 (2006) doi:10.1016/B0-08-044854-2/00497-1.

2. Halliday, M. A. K. & Hasan, R. *Cohesion in English*. (Routledge, London, 2013). doi:10.4324/9781315836010.

3. Ditman, T. & Kuperberg, G. R. Building coherence: A framework for exploring the breakdown of links across clause boundaries in schizophrenia. *J. Neurolinguistics* **23**, 254–269 (2010).

4. Taboada, M. & Mann, W. C. Applications of Rhetorical Structure Theory. *Discourse Stud.* **8**, 567–588 (2006).

5. Çokal, D. *et al.* The language profile of formal thought disorder. *NPJ Schizophr.* **4**, 18 (2018).

6. Huang, Y. *Pragmatics*. (Oxford University Press, Oxford, New York, 2015).

7. Kim, N., Brehm, L. & Yoshida, M. The online processing of noun phrase ellipsis and mechanisms of antecedent retrieval. *Lang. Cogn. Neurosci.* **34**, 190–213 (2019).

8. Hinzen, W. & Rosselló, J. The linguistics of schizophrenia: thought disturbance as language pathology across positive symptoms. *Front. Psychol.* **6**, (2015).

9. van Schuppen, L., van Krieken, K. & Sanders, J. Deictic Navigation Network: Linguistic Viewpoint Disturbances in Schizophrenia. *Front. Psychol.* **10**, (2019).

10. Elvevåg B, Foltz PW, Weinberger DR, & Goldberg TE. Quantifying incoherence in speech: an automated methodology and novel application to schizophrenia. *Schizophr Res* **93**, 304–16 (2007).

11. Morgan SE *et al.* Natural Language Processing markers in first episode psychosis and people at clinical high-risk. *Transl Psychiatry* **11**, 630 (2021).

12. Kuperberg, G. R. Language in schizophrenia Part 1: an Introduction. *Lang. Linguist. Compass* **4**, 576–589 (2010).

13. Andreasen, N. C. Scale for the assessment of thought, language, and communication (TLC). *Schizophr. Bull.* **12**, 473–482 (1986).

14. Arcara, G. & Bambini, V. A Test for the Assessment of Pragmatic Abilities and Cognitive Substrates (APACS): Normative Data and Psychometric Properties. *Front. Psychol.* **7**, (2016).

1. Patient population from studies with an Asterix have been pulled to account for the overall effect of SMIs (e.g. Schizophrenia and First Episode Psychosis were pooled and reported as a single score). [↑](#footnote-ref-1)
2. Expressive ragmatic components have been grouped under the main expressive pragmatic components included in this study. If different, exact wording used by author(s) are included in parenthesis. [↑](#footnote-ref-2)
3. "Discourse production is rated for the presence of communication difficulties at the contextual pragmatic level (e.g., over- or under-informativeness, abrupt topic shift) " [27, p. 108] [↑](#footnote-ref-3)
4. Average pairwise cosine similarity between a word and the 6^th^ following word. [↑](#footnote-ref-4)
5. Raw data provided upon contacting authors: Patients with Formal Thought Disorder: 0.24(0.06), Patients without Formal Thought Disorder: 0 .29(0.08). Patients’ data have been pooled. [↑](#footnote-ref-5)
6. Raw data provided upon contacting authors. [↑](#footnote-ref-6)
7. Raw data provided upon contacting authors: Patients with Formal Thought Disorder: 0.19(0.28), Patients without Formal Thought Disorder: 0 .04(0.09). Patients’ data have been pooled. [↑](#footnote-ref-7)
8. Raw data provided upon contacting authors. [↑](#footnote-ref-8)
